# Review of the Presence and Phage-Mediated Transfer of ARGs in Biofilms

**DOI:** 10.3390/microorganisms13050997

**Published:** 2025-04-26

**Authors:** Han Lu, Yanjun Wang, Hongyuan Liu, Nana Wang, Yan Zhang, Xinhua Li

**Affiliations:** 1 National Key Laboratory of Efficient Utilization of Nutrient Resources, Shandong Academy of Agricultural Sciences, Jinan 250100, China; lhan9408@163.com (H.L.);; 2 National Technological Innovation Center for Comprehensive Utilization of Saline-Alkali Land, Dongying 257347, China; 3 Shandong Provincial Key Laboratory of Water Pollution Control and Resource Reuse, School of Environmental Science and Engineering, Shandong University, Qingdao 266237, China

**Keywords:** antibiotic resistance genes, biofilm, phage-mediated transfer, resistance gene transfer

## Abstract

The widespread use of antibiotics has led to the emergence of a large number of drug-resistant bacteria, accelerating the dissemination and spread of antibiotic resistance genes (ARGs) in the environment. Bacterial biofilms, serving as reservoirs of ARGs, pose potential risks to environmental health that should not be ignored. Studies on the presence and transfer of ARGs in biofilms have been conducted both domestically and internationally. This article summarises the research progress on ARGs in various environments and analyses the mechanisms and factors influencing the dissemination and transfer of ARGs in microplastics, activated sludge, and pipe wall biofilms, with a particular focus on phage-mediated ARG transfer. We also discuss current research gaps in this field to provide references for future biofilm management and health risk control of ARGs.

## 1. Introduction

Antibiotics, one of the most important discoveries in medicine, play an important role in the treatment of bacterial diseases in humans and animals. With the widespread use of antibiotics, the problem of drug resistance has become increasingly prominent and poses a major health risk to human society. At present, there are at least 700,000 deaths per year worldwide due to antibiotic resistance, and if drug resistance is not effectively controlled, this number is expected to reach 10 million by 2050 [1]. Antibiotic resistance genes (ARGs) carried by clinically resistant pathogens usually have an environmental origin and are widely found in sewage, faeces, soil, and other media [2]. Therefore, ARGs have been recognised as emerging pollutants [3,4,5], and have become a research hotspot worldwide.

ARGs are prone to spreading and are widely distributed in the environment. In addition to water, atmosphere, and soil environments, ARGs are present in biofilms. Many resistant bacteria attach to biofilms on an object’s surface. These objects become “carriers” of the ARGs and have effects on subsequent gene transfer. Studies have shown that biofilms on the surface of microplastics (MPs) can promote the occurrence and dissemination of ARGs [6]. The presence of more microorganisms in biofilms provides convenient conditions for microbial interactions with a large enrichment of ARGs and mobile genetic elements (MGEs) on the surface, which also strengthens horizontal gene transfer (HGT) and quorum sensing (QS) of ARGs [7].

ARGs undergo horizontal transfer, enter the body through the food chain, and pose a threat to human health. Among them, phages in the environment can induce the horizontal transfer of ARGs through transduction, increasing the risk of ARGs spreading [8]. It has been confirmed that phage genomes extracted from different environmental samples carry ARGs and can be used as reservoirs of ARGs in the environment, such as in urban sewage and rivers, effluent from treated municipal sewage and medical wastewater, and activated sludge [9,10,11]. However, the influence of phages on the horizontal transfer of ARGs in biofilm systems remains unclear, and the specific mechanisms of their roles in biofilm formation and drug resistance have not been fully studied.

In recent years, studies on ARG pollution have increased; however, most have focused on water and soil environments. There have been few studies on ARGs in biofilms. In addition, substances coexisting with ARGs and the physical and chemical properties of the environment can affect the transmission of ARGs. The factors influencing the spread of ARGs in different environments are still under investigation. Therefore, this paper reviews the spread of ARGs in the environment in combination with current research hotspots, with a focus on the ARGs in biofilms of pipelines, MPs, and activated sludge, and summarises the research progress of phage-mediated ARG transfer in biofilms. The aim is to fill the relative gaps in the research on ARGs in the biofilm environments and provide a basis for the study of the regulatory mechanisms underlying phage-mediated ARG transfer, biofilm formation, and drug resistance.

## 2. Transmission and Diffusion of ARGs in the Environment

### 2.1. ARGs in the Environment

The presence of ARGs, considered emerging contaminants, in the environment is characterised by widespread occurrence, persistence, replicability, and mobility. The main pollution sources of ARGs include hospitals, animal husbandry, and wastewater from urban sewage treatment plants. The environment can serve as the main repository of ARGs, storing ARGs from animal and human sources, while also acting as a pollution source to transmit ARGs to the human environment. ARGs can migrate and transform in different environmental media and have been found in soil, water, and the atmosphere. They can also invade the human body through the food chain, potentially causing harm to human health [12]. Soil is one of the largest receptors of antibiotics and ARGs, and the application of manure to breeding farms provides access to the soil environment. Most ARGs have been detected because of their abundance in soil [13]. For example, more than 40 tetracycline-resistance genes have been detected, of which *tetA*, *tetO*, *tetW*, and *tetZ* are the most common [14]. Alternatively, abundant microorganisms in the soil provide a vehicle for the horizontal transmission of ARGs. The ARGs can accumulate through the “soil–microbe–plant–animal” transmission chain, which not only seriously affects ecological security but also endangers the upper food chain and thus threatens human health [15]. Aarestrup et al. [16] found that *Enterococcus faecalis* isolated from chickens, pigs, and humans had a similar drug-resistance spectrum and resistance genes, which indicated that the daily diet is a potential way for ARGs to enter the human body. It also proved that ARGs could harm human health through the enrichment and accumulation of soil ecosystems as well as the transmission of food chains.

As an important reservoir and diffusion channel for ARGs, the water environment can spread point-source-contaminated ARGs to a larger environment, resulting in further contamination of water bodies with ARGs. Currently, ARGs have been detected in the water environments of groundwater, ocean, lakes, reservoirs, and rivers. Groundwater is relatively less contaminated by antibiotics and resistance genes; however, the contamination of groundwater by ARGs has gradually attracted attention, with the detection of more ARGs in groundwater. Szekeres et al. [17] investigated the presence and prevalence of ARGs in groundwater in Cluj, Romania. The results showed that ARGs were prevalent in the surveyed groundwater environments and that the abundance of ARGs increased not only in urban areas but also in remote areas. Currently, research on ARGs in groundwater is limited, both domestically and internationally. However, the infiltration of surface pollutants and low-temperature dark conditions inherent to groundwater are conducive to the storage of ARGs [18]. Therefore, groundwater is likely an important reservoir of ARGs. Owing to the rise in aquaculture and the influx of terrestrial runoff, ARGs are frequently detected in the ocean and are found in coastal, deep-sea, and polar waters [19,20,21]. Moreover, with rapid economic development, industrial, agricultural, and aquacultural activities in lake basins have expanded significantly, leading to a further increase in antibiotic use and pollution in lake basins. In addition, the slow hydraulic circulation in lakes allows pollutants to remain in the water for extended periods, making lakes more prone to storage and accumulation of ARGs [22]. Analysis of ARGs in 15 lakes in the middle and lower reaches of the Yangtze River showed that sulphonamide ARGs were dominant in the lakes [23]. Compared with lakes, reservoir systems are more strictly controlled and less polluted with antibiotics and ARGs; however, contamination with ARGs still exists. Dang et al. detected 436 ARG subtypes in 20 resistant genotypes, including macrolides, sulphonamides, and aminoglycosides, in the Danjiangkou Reservoir, the second-largest reservoir in China [24]. Zhang et al. studied the contamination levels of 19 ARGs in water supply reservoirs in Central China, and the detection rate of 7 ARGs (*sulI*, *sulII*, *qnr*S, *strA*, *strB*, *blaAM*P, *blaTEM-1*) was 100%, but both their relative and absolute abundances were found to be at relatively low levels [25]. River water environments are significantly affected by human activities, and the detection rate and abundance of ARGs are relatively high. Nnadozie et al. performed a statistical analysis of the detection rate of ARGs in rivers and lakes in many places. The results showed that the highest detection rate in water was in rivers (98%), followed by lakes (77%), and the lowest detection rate was in reservoirs (<1%) [26].

Resistant bacteria in sewage and sludge from different wastewater treatment units can enter the air environment through various mechanisms (evaporation, aeration, stirring, etc.) [27]; therefore, strains carrying ARGs are commonly found in the air environment of sewage treatment plants. Relevant studies have detected resistance genes in the air around operational treatment units such as aeration tanks, settling tanks, anaerobic tanks, and sludge dewatering rooms, as well as in nearby residential areas. Li et al. used a SKC Biosampler (SKC, Inc.), a liquid impinger, to collect air samples in 20 mL deionised (DI) water from both the screen room and the bioreactor basin, in which *sulII* and class I integrases were detected [28]. Wang et al. indicated that the concentrations of *blaTEM-1*, *blaOXA-1*, and *blaAmpc* in the onboard near the sludge concentrator were 2.6 × 10^4^ copies/m^3^, 4.8 × 10^3^ copies/m^3^ and 8.8 × 10^2^ copies/m^3^, respectively [29]. High levels of ARGs have also been detected in the air environments of livestock farms. Many studies have shown that the prevalence of tetracycline- and sulphonamide-resistance genes in livestock farms is related to the extensive use of these drugs [30,31]. Hospitals are also places with high antibiotic usage, where ARGs are commonly found. Hospitals have limited per capita space and high population density, which facilitate the rapid spread and dissemination of bacteria carrying resistance genes. Wang et al. detected 19 resistance genes in the air environment of a hospital [29]. Floors, sheets, patients, and external environments within some wards may also be sources of resistance genes. Gilbert et al. showed that *ermX* genes and some tetracycline-resistance genes in ward air were also derived from these environments [32]. Moreover, Shiomori et al. suggested that *Staphylococcus aureus* carrying resistance genes in the air might also originate from this [33]. ARGs in the air can enter the human body by attaching to atmospheric particulate matter. In conclusion, ARGs are emerging pollutants in various environments that pose a significant threat to human health.

### 2.2. Transmission of ARGs

The transmission of ARGs mainly occurs through vertical and horizontal transfer. Vertical gene transfer (VGT) refers to the parental inheritance of resistance genes in offspring during the reproductive process of bacterial division and proliferation. HGT liberates genes from normal vertical inheritance, allowing microorganisms to acquire new genetic material from outside their clonal lineages. This enables ARGs to spread between bacteria that are not in a parent–offspring relationship, making gene flow more complex [34]. When bacteria are under selective pressure from antibiotics, the horizontal transfer of ARGs can provide them with the genes needed for survival faster than spontaneous mutations, allowing them to rapidly acquire the ability to adapt to a new environment [35].

**Figure 1:**
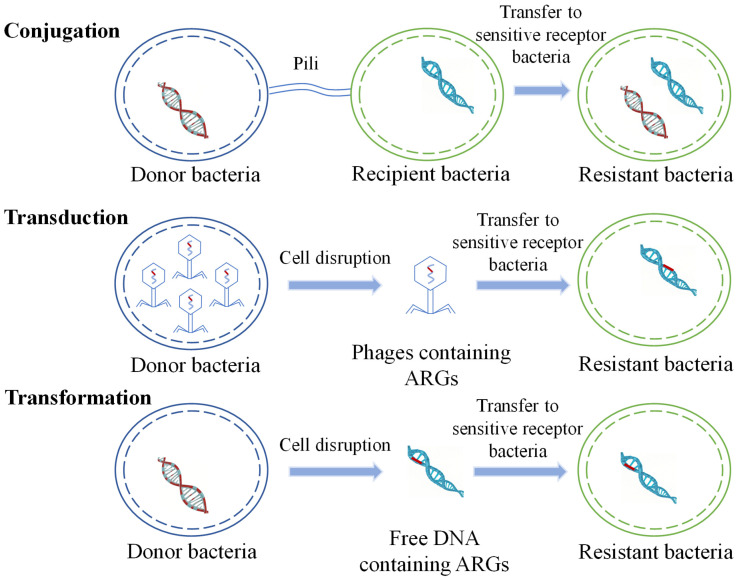
Schematic diagram of horizontal transfer of ARGs.

HGT is considered to be the main route for the spread of ARGs in the environment and plays a crucial role in the transmission of antibiotic resistance. ARGs are typically contained in mobile gene elements (MGEs), including plasmids, transposons, and integrons. HGT occurs via transduction, transformation, and conjugation (Figure 1) [36,37,38,39,40].

Regardless of whether antibiotics are used to treat diseases or are added to animal feed, their entry into humans or animals induces the production of resistant bacteria and resistance genes. After resistant bacteria and resistance genes from livestock, animal faeces, human faeces, urine, industrial wastewater, and domestic sewage enter the environment, they undergo gene exchange with indigenous bacteria through HGT. Resistance genes, due to rainfall or surface runoff, can migrate in soil and water environments, leading to the contamination of water environments with antibiotic-resistant bacteria (ARB) and ARGs. In contaminated soil, the horizontal transfer of ARB and ARGs may also occur between the soil and crops. Plant- and animal-based edible products from livestock and poultry enter the human body through the food chain, where they can accumulate and pose potential health risks.

### 2.3. Factors Affecting ARGs Transfer

#### 2.3.1. Antibiotic Selection Pressure

Antibiotics are widely used to prevent human diseases caused by bacterial infections and to promote the growth of livestock and poultry on livestock farms. However, with the widespread use of antibiotics, their residual levels in the environment have gradually increased, creating selective pressure on bacteria, which, in turn, promotes the production of antibiotic-resistant bacteria and ARGs [41,42,43]. The development of antimicrobial resistance (AMR) can lead to the acquisition of resistance genes through mutations or HGT, with the latter considered the most important factor in the current AMR epidemic.

Most antibiotics are excreted in a prototype form and enter the environment, either directly or through liquid waste. ARGs and MGEs can spread to symbiotic bacteria and pathogens under antimicrobial selection pressure [44]. Larsson et al. [45] showed that when water was taken from a pharmaceutical production site of sewage treatment plants in India, very high levels of antibiotics were found in the surface layers, ground, and drinking water. Increased antibiotic selection pressure has altered bacterial HGT pathways [46], making multi-resistant strains common not only in treatment plants but also in nearby river sediments with high levels of ARGs and MGEs.

In addition to the detection of resistance genomes in natural environments, the gut flora of human and animal species can clarify whether clinically relevant ARGs are widely present in the monitored environment [47,48]. For example, the plasmid-mediated polymyxin resistance gene MCR-1 (mobilised colistin resistance gene 1), mined from a metagenomics database, was recently found in the intestinal pathogens and symbiotic strains of clinical patients in China, indicating that this gene has emerged and spread in human intestinal microbes [49]. A growing number of studies have revealed that many unknown ARGs occur in soil or activated sludge, as well as in animal and human flora [50,51]. Studies have also demonstrated that ARGs are mainly clustered in ecology, indicating that drug-resistant genomes in soil and sewage treatment plants are significantly different from those of human pathogens [52]. However, researchers have found that some resistance genomes are similar. This suggests that selective pressure for increasing antibiotic use leads to the generation and spread of bacterial resistance and highlights the important role of HGT in pathogen resistance to antibiotics.

#### 2.3.2. Heavy Metals

Recent studies have shown that antibiotics are not the only factor inducing the generation of ARGs [53]. (The study searched for relevant English-language literature published in databases such as Web of Science, ScienceDirect, and Scopus between 2015 and 2025. The search keywords included “antibiotic resistance”, “ARGs AND generation OR dissemination OR transmission”, “factors”, etc. Articles discussing the influencing factors of ARGs generation and transmission were included, while irrelevant literature, conference abstracts, and informal publications were excluded.) Heavy metals play a significant role in the generation and dissemination of ARGs. They can exert selective pressures on the environment, forcing microbial communities to adapt and survive. This adaptation involves not only the development of resistance to the heavy metals themselves but also the acquisition and transmission of ARGs through HGT mechanisms. Cu^2+^ and Zn^2+^ are two common heavy metals in water bodies that are widely used together with antibiotics in animal husbandry to reduce diseases and promote growth [54]. However, only a small portion is absorbed and utilised, most of which is excreted in the faeces, leading to elevated levels of heavy metals in livestock manure. It has been shown that sub-inhibitory levels of antibiotics and heavy metals can promote the horizontal transfer of ARGs between bacteria. The long-term presence of heavy metals in the natural environment has a more profound impact on the transfer of ARGs than antibiotics and organic compounds, as antibiotics and organic matter are naturally degraded by the ecosystem over time, whereas heavy metals are not easily degraded. Therefore, their impact on microorganisms persists for a long time. Although heavy metals naturally exist in the environment, their presence has significantly increased in recent years owing to their inclusion in fertilisers and animal feed. Moreover, the discharge of wastewater from livestock farming into the environment has led to a substantial increase in heavy metal concentrations in water bodies and other ecosystems [55,56]. Sewage treatment plants, landfills, agriculture, hospitals, and other sites, along with environmental pollution and human activities, also contribute to the increase of heavy metal content in the environment, thereby affecting the transfer of ARGs. Industrial wastewater, agricultural fertilisers, mining activities, and other factors often lead to heavy metal pollution. Especially in areas with high concentrations of heavy metals in water and soil, bacteria tend to develop resistance mechanisms to cope with these pollutants, and these mechanisms are sometimes associated with ARGs. Li et al. found that bacterial conjugation frequencies in groups co-stressed with Hg^2+^, Cu^2+^, and Zn^2+^ increased by 2.57, 1.60, and 1.74 times, compared to the control group, when exposed to cefotaxime sodium [57]. Zhang et al. showed that sub-inhibitory concentrations of heavy metals such as Ag (I) and Cr (VI) could promote the intra-species conjugation transfer of ARGs in *Escherichia coli* [58]. In a study conducted by Jesse et al. on soil pollutants at the Savannah River site in South Carolina, USA, the abundance of ARGs was high in areas with high heavy metal concentrations [59]. Knapp et al. found a significant correlation between soil Cu content and the abundance of five ARGs (*tetW*, *tetM*, *blaOXA*, *ermB*, and *ermF*) and soil Cr content with *tetM*, *blaCTX-M*, and *blaOXA* abundance [60]. Devarajan et al. showed that heavy metal concentrations in sediments were significantly and positively correlated with ARG abundance in Lake Geneva, Switzerland [61]. This suggests that the propagation of ARGs is influenced by a combination of heavy metals and mobile elements.

The mechanisms by which heavy metals affect bacteria mainly include oxidative stress, interference with cellular metabolism, damage to cell structure, induction of genetic mutations, and activation of response mechanisms. Heavy metals such as Cu, Zn, Pb, and Hg can increase bacterial oxidative stress by generating free radicals and reactive oxygen species (ROS), leading to damage to the cell membrane and DNA, which in turn affects bacterial growth and survival. Heavy metals can directly bind to bacterial enzymes, proteins, and other important biological molecules, inhibiting their normal metabolic processes. For example, heavy metals may interfere with the bacterial respiratory chain, protein synthesis, and energy metabolism. Additionally, the accumulation of heavy metals can disrupt the integrity of the cell membrane, causing changes in membrane permeability and affecting the function and structure of the cell. Their accumulation within bacteria can lead to genetic mutations, thereby altering the genetic characteristics of the bacteria, and sometimes, these mutations may result in the bacteria acquiring drug resistance. Furthermore, bacteria can respond to the toxicity of heavy metals through specific response mechanisms, such as metal stress responses. Some bacteria reduce the accumulation of heavy metals by secreting metal ion efflux pumps or mitigate the harm of heavy metals by altering metabolic pathways.

Although heavy metals are important factors that promote the horizontal transfer of ARGs, the molecular-level regulatory mechanisms controlling this process in bacteria are not yet fully understood. Most studies have focused on analysing the mechanisms of ARG transfer only after conjugative transfer is completed in pure bacterial cultures, which may overlook changes in regulatory genes during the conjugation process. Gene expression and abundance within bacteria can change rapidly; some key regulatory genes may be overexpressed and begin to function during the conjugative transfer process, but their expression levels may decrease after the transfer reaction occurs. Therefore, studying the molecular mechanisms of the horizontal transfer of ARGs under common Cu^2+^ and Zn^2+^ concentrations is of significant importance for developing advanced biotechnologies to target and control the spread of ARGs in the environment.

#### 2.3.3. Nanomaterials

In recent years, nanomaterials have been widely applied in agriculture, medicine, and industry owing to their advantages such as large specific surface area and excellent anti-bacterial properties. However, nanomaterials can promote the horizontal transfer of ARGs, thereby increasing the risk of dissemination of bacterial resistance in the environment; therefore, the rational use of nanomaterials has attracted widespread attention.

Nanometal particles can affect ARG abundance and diversity in complex environments. Ji et al. used metagenomic sequencing to analyse the impact of copper nanocomposites on ARGs, and their results showed that the presence of copper nanocomposites significantly reduced the abundance of ARGs [62]. In their study of the effects of different metal nanomaterials on microbial communities, Su et al. found that metal nanomaterials can significantly reduce the abundance and diversity of ARGs [63]. In addition to directly affecting the abundance and diversity of ARGs, nanometal ions can influence the HGT of ARGs. Studies have shown that HGT can be promoted by conjugation [64]. Pu et al. found that simultaneous exposure to cadmium and nano-Fe_2_O_3_ significantly increased the transfer of the RP4 plasmid from *E. coli* to aquatic bacterial communities [65]. Moreover, metal nanoparticles can bind to DNA, and after binding, they can serve as carriers for the transfer of ARGs to the cell surface or into the cell, thereby accelerating HGT.

**Figure 2:**
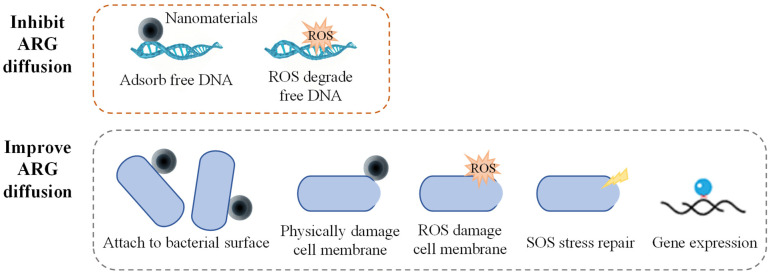
Mechanisms of nanomaterials on ARG diffusion.

The mechanisms through which nanoparticles influence the diffusion of ARGs are primarily related to the impact of the physical properties of the nanoparticles on the physical state of bacteria, as well as the regulation of bacterial physiological metabolism by the ROS generated by the nanoparticles (Figure 2). Most nanomaterials promote the diffusion of ARGs in pure bacterial systems. Qiu et al. studied the effects of factors, such as the concentration of nano-Al (O), bacterial conjugation density, conjugation time, and temperature, on the conjugative transfer of ARGs. The results showed that under optimal conditions, nano-Al_2_O_3_ increased the inter-genus transfer efficiency of the RP4 plasmid by more than 200 times and the intra-genus transfer efficiency by more than 250 times [66]. Ding et al. also found that nano-Al_2_O_3_ could significantly promote the inter-genus transfer of antibiotic-resistant plasmids carried by the Gram-negative bacterium *E. coli* to the Gram-positive bacterium *S. aureus* [67]. Graphene oxide (GO) has a minimal impact on antibiotic-resistant bacterial activity. High concentrations of GO (>10 mg·L^−1^) could damage the resistance plasmids and reduce bacterial resistance to antibiotics, while low concentrations of GO (<1 mg·L^−1^) caused almost no damage to plasmids. All experimental concentrations of GO significantly enhanced the ARG transfer efficiency. These results suggest that the mechanical damage caused by insoluble nanomaterials imposes relatively little stress on the bacteria [68]. Nanomaterials can release toxic heavy metal ions and have high microbial toxicity; their promoting effect on ARG transfer is only reflected under low-stress conditions. Wang et al. found that bacterial exposure to sub-lethal concentrations (1–10 mg·L^−1^) of ZnO nanoparticles significantly increased the conjugation frequency of the antibiotic resistance plasmid RP4. Among them, the conjugation transfer efficiency between *E. coli* increased by 24.3 times, while the horizontal transfer efficiency of the mixed microbial communities increased by 8.3 times. Furthermore, nano-ZnO boosts by three times the transformation efficiency of *E. coli* by acquiring the naked plasmid pGEX4T-1 [69]. In addition to the adsorption of nano-ZnO, the reduction of its toxic effects on cell membrane stability may also play an important role in promoting plasmid transformation.

The actual environmental conditions are complex, and microorganisms usually exist in the form of mixed microflora; therefore, the influence of nanomaterials on the transmission of ARGs in the actual environment is more complex. Zou et al. studied the effects of the coexistence of GO and antibiotics on the bacterial uptake of antibiotics and ARGs in lake water [70]. The results showed that GO could form a complex with extracellular antibiotics, thereby inhibiting bacterial antibiotic uptake and reducing the abundance of ARGs by two to three orders of magnitude. GO can reduce the transfer efficiency of integrons carrying ARGs by 55 times under optimal conditions. Huang et al. studied the effects of CuO and ZnO nanoparticles on ARG proliferation during the anaerobic digestion of sludge [71]. These results showed that CuO and ZnO promoted the environmental transmission of *tetC*, *tetQ*, *sulI*, and *sulII*. These findings (Table 1) suggest that the role of nanomaterials in ARG diffusion may involve multiple factors and mechanisms, with the final impact potentially dependent on the combined effects of multifactorial synergies.

## 3. ARGs in Biofilms

Because most bacteria in nature live in biofilms, it seems reasonable that HGT occurs more frequently in biofilms than in planktonic cells. In recent years, biofilms have been reported as repositories [72,73,74].

### 3.1. MP Biofilms

Plastic products are widely used in various fields worldwide, and plastic production is steadily increasing by 300 million tons annually. Plastic waste can persist in the natural environment for extended periods and break down into smaller fragments through physical, chemical, and biological degradation processes. MPs are particles or fragments smaller than 5 mm that can accumulate in living organisms and can be transferred through the food chain, posing a potential threat to human health [75]. Because of the non-degradable nature and hydrophobic surface of plastics, they easily absorb harmful substances from the environment, acting as carriers for the long-term accumulation and transfer of organic compounds and chemicals (such as polycyclic aromatic hydrocarbons, antibiotics, and metals, etc.) [76,77,78].

**Figure 3:**
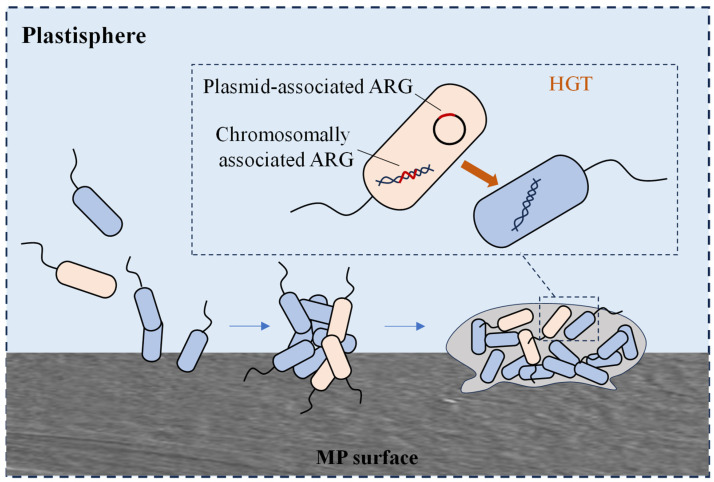
HGT of plasmid-mediated ARGs occurring between planktonic bacteria in the plastisphere.

Microorganisms can attach to the surface of MPs and survive by utilising the hydrophobic substances adsorbed on the plastic surface as nutrients. These microorganisms use MPs as carriers to form biofilms on their surface [79]. The microbial community in MP biofilms differs significantly from that in the surrounding environment or nonplastic substrates, with certain potential pathogens selectively enriched in MP biofilms. Microbe-colonising MPs in the ocean differ from those in the surrounding water. Human pathogenic *Vibrio* spp. were detected in biofilms on the surfaces of polyethylene (PE), polypropylene (PP), and polystyrene (PS) particles. Additionally, the potential pathogen *Pseudomonas* is more abundant on MP surfaces than on nonplastic surfaces [80,81]. In addition, the diversity and abundance of bacterial ARGs in MP biofilms and other substrate biofilms were significantly higher than those of free bacteria in water, which may be related to the high bacterial density, close cell-to-cell contact within the biofilms, and the accumulation and enhancement of MGEs in the MP microenvironment (Figure 3). Oberbeckmann et al. found that the abundance of ARB *Sphingopyxis* was significantly higher in MP biofilms [82]. MPs may serve as hotspots for the transport and transfer of potentially pathogenic bacteria and antibiotic resistance.

The community structure and diversity of MP biofilms are influenced by various factors, such as water source, plastic type, plastic size, and seasonal variations. Research by Parrish et al. indicated that the water source and plastic type were the primary drivers of the bacterial community structure in MPs biofilms. At the same time, *sulI* and potential pathogens (e.g., *Pseudomonas*) were detected in the biofilm, suggesting that MPs may have the potential to carry ARGs [83]. MPs, as unique microbial habitats, can alter the microbial community structure, thereby potentially affecting the ecological functions of microbial communities in aquatic ecosystems. It is evident that MPs serve as carriers for microorganisms, harbouring specific bacterial communities, including a high abundance of potential pathogens.

Currently, the research on ARGs in MP biofilms is limited. Biofilms can protect bacteria, especially in adverse environments such as those contaminated with antibiotics, metals, pesticides, and other pollutants. Owing to the high cell density within biofilms, intercellular interactions are more frequent, promoting the accumulation of ARGs and metal resistance genes (MRGs). In such environments, QS communication and genetic transfer via HGT have become more efficient [84]. When bacterial communities in biofilms are exposed to low (below sub-inhibitory) concentrations of chemicals and constant chemicals (such as antibiotics), these pollutants alter the behaviour of bacteria to varying extents, potentially accelerating HGT and the spread of ARGs across a wide range of bacterial species.

MP biofilms are unique microbial habitats that selectively enrich ARGs, MRGs, and pathogenic bacteria. As a result, MPs are considered reservoirs for ARGs and MRGs, as well as potential vectors for the transmission of pathogenic bacteria. Currently, research on MPs and ARGs has mainly focused on the distribution of MPs and the abundance characteristics of ARGs in specific environments (such as oceans, farms, and wastewater treatment plants). However, ARGs in MP biofilms, bacterial community structures, and their interactions under antibiotic and metal selection pressures require further in-depth studies.

### 3.2. Activated Sludge Biofilms

Various types of wastewater that may carry a large number of antibiotic-resistant microorganisms and resistance genes, such as medical, livestock breeding, and antibiotic industrial wastewater, ultimately flow into sewage treatment plants. Therefore, sewage treatment systems are considered sources of ARGs. As most sewage treatment plants use biological treatment processes based on activated sludge, the interaction between microorganisms and antibiotics in activated sludge has sparked intense discussion among researchers.

Sludge is an important by-product of sewage treatment plants and cannot be overlooked. Research on ARGs in sludge has begun. Ma et al. detected multiple ARGs in sludge samples from a sewage treatment plant in the United States. The absolute concentration of the nine tested tetracycline-resistance genes ranged from 10^8^ to 10^10^ [85]. Munir et al. conducted quantitative PCR detection of ARGs in effluents and sludge from sewage treatment plants using different treatment technologies. They concluded that most ARGs carried by the influent water were concentrated in the sludge after passing through the treatment system. The concentration of ARGs in the effluent entering the environment is significantly lower than the number of resistance genes retained in the sludge and discharge [86]. Additionally, the presence of antibiotics can increase bacterial resistance in the sludge system, which in turn reduces the ability of the system to effectively remove pollutants [87]. Zhang et al. also found that environmental concentrations of tetracycline (0–500 µg/L) altered the microbial community in aerobic granular sludge (AGS), enriching the sludge with ARGs [88]. Antibiotic pressure also has an impact on the cultivation. Some studies have shown that biofilm formation under antibiotic pressure is a defence mechanism that helps microorganisms resist the harmful effects of antibiotics and provides a protective environment that supports ARB survival [89]. They observed that activated sludge exhibited significant aggregation behaviour in the presence of tetracycline [90]. AGS is a special type of biofilm formed by microbial aggregation.

**Figure 4:**
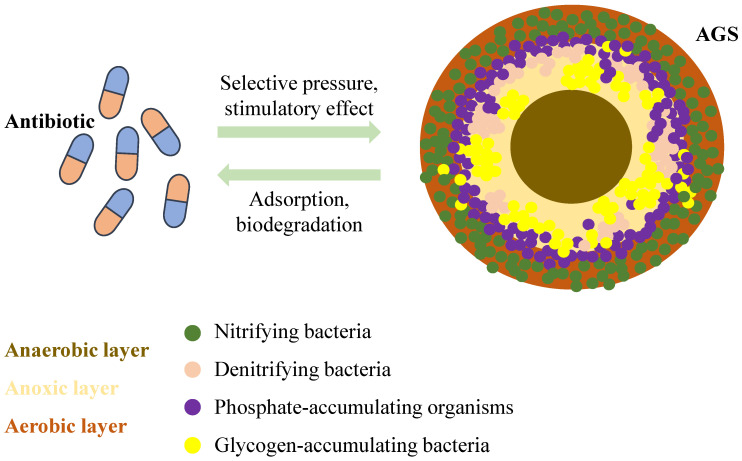
Mechanism of action between antibiotics and AGS.

AGS is a granular structure that resembles a sphere formed from regular activated sludge. It is generally considered to be AGS when the particle size of these aggregates reaches 200 µm [91]. Its outer surface is yellowish-brown or light yellow, and its interior is divided into three distinct zones: aerobic, anoxic, and anaerobic layers, which are composed of microorganisms, extracellular polymeric substances (EPS), and other deposits, respectively (Figure 4). AGS has a high concentration and rich diversity of microorganisms, including various aerobic microorganisms, anaerobic microorganisms, and animals. Antibiotics can induce biofilm formation on sludge surfaces. Simultaneously, the dense biofilm structure formed by the AGS may provide an excellent environment for ARG transmission. However, no studies have systematically analysed the fate of resistance genes during the formation of AGS under environmentally relevant concentrations of antibiotics. Nevertheless, there was a correlation between the QS system, AGS, and ARGs.

Microbial aggregates are formed under the influence of various environmental factors, such as nutrient concentration, oxygen levels, temperature, changes in chemical substances, and predation pressure. An increasing number of studies have shown that QS plays a significant role in particle formation and stabilisation. There are more signalling molecules (e.g., Autoinducers, AIs) in mature particles than in early or smaller particles [92,93]. Moreover, as the production of signal molecules increases during the AGS granulation process, the biofilm development rate is ten times higher than that in the initial stage, strongly suggesting a strong correlation between QS signals and the granulation process. Recent studies have suggested that AI-2 can affect aerobic granulation through EPS secretion and enhance bacterial adhesion [94]. In addition, decreased concentrations of acyl homoserine lactones (AHLs) lead to the disintegration of particles or smaller particle sizes [95]. Overall, the QS system had significant effects on granulation through several factors such as EPS production, changes in microbial community composition, and AHL concentration. Further studies revealed that the QS mechanism can regulate the expression of bacterial genes, including bacterial drug resistance and virulence determinants. Bjarnsholt et al. found in their study on the QS system of *Pseudomonas aeruginosa* that bacteria in the experimental group, where the QS system was inhibited, were unable to survive in the presence of tobramycin and H_2_O_2_. However, *P. aeruginosa*, with a functional QS system, is able to survive. Furthermore, inhibition of the QS system did not significantly affect normal bacterial growth. Experimental results have demonstrated that microbial QS systems play a vital role in antibiotic resistance regulation [96]. QS influences bacterial antibiotic resistance by regulating the expression of membrane proteins. Disruption of QS activity can negatively affect the transmission of ARGs in the environment [97]. Biofilms are ubiquitous in aquatic environments and facilitate the acquisition and spread of ARGs. They also serve as ideal sites for ARG exchange. Zhu et al. studied the effect of QS on the horizontal transfer of the multi-drug plasmid RP4 in biologically activated carbon (BAC) biofilms and found that AHLs (e.g., C_8_-HSL) and AHL-producing bacteria isolated from BAC biofilms affected the horizontal transfer of ARGs [98]. The granular process of AGS is a process of biofilm formation, which may play a key role in increasing and spreading antibiotic resistance in bacteria; however, this aspect has not been explored.

### 3.3. Pipe Wall Biofilms

**Figure 5:**
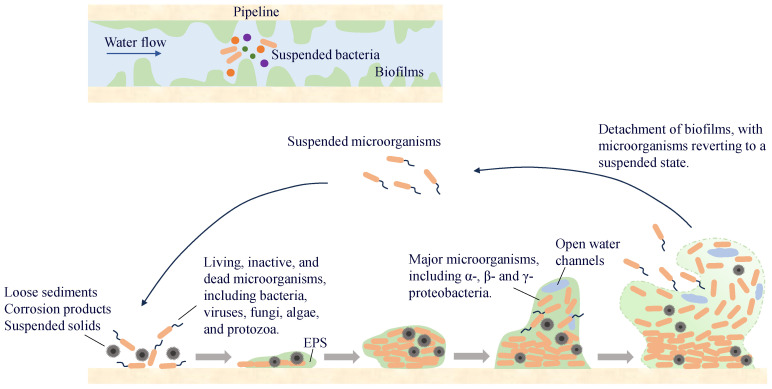
The process of biofilm formation.

A biofilm on a pipe wall is a biological film that attaches to the wall surface and is composed of microorganisms, secreted EPS, and inorganic materials. More than 90% of the microorganisms in the water-supply network exist in the pipe-wall biofilm, whereas the proportion of microorganisms in the water is relatively small. Microorganisms in the water exist in suspension and migrate in the pipeline with the water flow. When environmental conditions are suitable, they attach to the wall surface and enter a reversible attachment phase, during which they undergo adsorption and detachment from the surface. As bacteria continue to proliferate and differentiate, especially during the synthesis of EPS, microorganisms become encapsulated within them and gradually fix to the pipe wall surface, entering the irreversible attachment phase. With the synthesis of QS molecules, microorganisms proliferate and differentiate to form multi-layered cell structures, which ultimately develop into stable biofilms. When environmental factors change, microorganisms in the biofilm detach and enter water (Figure 5). Some of them will migrate and form new biofilms at other locations in the pipeline, whereas others are carried by the water flow to the end of the water supply system, posing potential microbial safety risks.

The unique structure of biofilms provides microorganisms with a conducive environment for their survival and protection. Studies have shown that chlorine has a weak penetrating effect on biofilms and must react with EPS before it can interact with microorganisms [99]. The framework formed by inorganic materials and EPS within the biofilm helps maintain its stability under the sheer force of the water flow. Compared with suspended microorganisms, those in biofilms are less susceptible to predation by protozoa in water [100]. Owing to the protective nature of biofilms, microorganisms within them are more likely to survive in the water supply network, making biofilms an important reservoir of microbial genetic material in these systems [101].

#### 3.3.1. Factors Influencing Biofilm Formation

Biofilm formation on pipe walls is influenced by various factors, including nutrient conditions, temperature, pipe material, hydraulic conditions, and operational parameters. Microorganisms in pipe-wall biofilms utilise biodegradable organic matter (BOM) through dissimilatory processes to synthesise cellular components. Oxidation processes, such as ozone treatment, ultraviolet (UV) treatment, and UV/H_2_O_2_ advanced oxidation, can convert macromolecular organic compounds in water into small, biodegradable substances that may serve as nutrients for microbial growth in biofilms on pipe walls. Inorganic substances present in the water-supply network, such as nitrogen, phosphorus, and trace metal elements, also influence biofilm growth. Zhang et al. [102] found that changes in the ratio of nutrients (such as carbon and nitrogen) could alter the composition of bacterial communities in biofilms. Specifically, environments with high nitrogen/carbon ratios are more favourable for the growth of autotrophic bacteria, whereas environments with low nitrogen/carbon ratios promote the growth of heterotrophic bacteria. To prevent the corrosion and aging of pipes due to prolonged use, countries such as the United Kingdom and the United States typically add an appropriate amount of phosphate to treated water. This is because phosphate can form stable complexes with the surfaces of corroded metal pipes, effectively reducing further damage to the pipes [103]. Fang et al. [104] found that the addition of phosphate led to the formation of thicker biofilms with greater heterogeneity, while also enriching the diversity of microbial species. Therefore, a moderate change in phosphorus content significantly altered the structure of the biofilm and the composition of the bacterial community.

Although the water supply network was buried underground, it was still affected by external temperature fluctuations. Variations in climate temperature can influence the initial adhesion process of bacteria, as well as the formation of biofilms. Temperature can also affect the expression of certain genes, thereby influencing the secretion of microbial EPS and the hydrophobicity of bacterial surfaces. This may be because bacteria adjust the lipid composition of their cell membranes in response to temperature changes, altering the hydrophobicity of the membrane and ultimately affecting its adsorption capacity on a specific surface [105]. Although low temperatures can slow down the bacterial growth rate, Labidi’s research indicated that *Listeria monocytogenes* strains were still capable of forming biofilms even at 1 °C [106].

Commonly used pipe materials in water-supply networks include cast iron, steel, copper, concrete, and plastic (such as polyvinyl chloride (PVC) and PE). The selection of pipe materials affects the formation and characteristics of biofilms on the pipe walls. Plastic pipes are a source of biodegradable volatile organic compounds in drinking water. During water transportation, phenomena such as the leaching of polymer additives, degradation of high polymers, and generation of by-products from polymer oxidation may occur [107]. Studies have shown that microorganisms can utilise low-molecular-weight plasticisers, residual polymer monomers, and antioxidants for proliferation, thereby promoting biofilm growth in water supply networks [108]. However, some researchers found that the microbial community diversity of biofilms on plastic pipes was lower than that on metal pipes [109]. The inner wall of the plastic pipe was relatively smooth, whereas the inner wall of the metal pipe was relatively rough owing to corrosion and other factors after a period of operation, which can better protect microorganisms from the influence of hydraulic shock and disinfectants. In addition, substances such as scale and rust on the inner surfaces of metal pipes increase the contact surface area, promoting the attachment and growth of microorganisms. Simultaneously, the dissolved and suspended corrosion products contribute to the proliferation of specific biofilm microbial communities.

Hydraulic conditions significantly affect biofilm formation, leading to structural differences. During the initial stages of cell attachment and biofilm formation, high flow promotes the transfer of bacteria from water to the biofilm [110]. Subsequently, in the already-formed biofilms, the high shear forces generated by the high flow stimulated the synthesis of EPS and enhanced bacterial attachment to the carrier surface. These EPS further improve the mechanical stability of the growing biofilm, helping bacteria in the water to adhere to the pipe walls. Simultaneously, high-flow water accelerates the exchange of materials between the water and biofilm, promoting the uptake of nutrients by the biofilm, thereby accelerating the growth of microorganisms within the biofilm [111]. By contrast, high-flow water accelerates biofilm detachment [112]. The shed biofilms carry enriched microorganisms that may deteriorate the quality of drinking water and threaten human health. In contrast, under low-flow conditions, the material exchange rate between the biofilm and water was lower, resulting in a looser and more porous biofilm structure.

The operational conditions of a water-supply network have a significant impact on the growth of pipe-wall biofilms. Water consumption in urban supply systems follows a periodic pattern, with significant differences in water usage and pipeline flow rates between peak and off-peak periods. Considering the impact of water consumption, Fish et al. designed a model to simulate variations in the inflow rates of water supply networks and found that as flow rate fluctuations increased, the biomass within the biofilm increased; however, both the EPS/bacterial ratio and microbial diversity showed a declining trend [113]. Under peak operating conditions, there are significant flow fluctuations, and the exchange of biofilms and substances in the water is enhanced, which is conducive to the acquisition of nutrients by bacteria in the biofilm, promoting their growth and reproduction. However, studies have shown that intense shear force fluctuations also prompt bacteria to adapt to suspension growth to detach from the biofilm and enter water, accelerating the process of biofilm shedding [114].

#### 3.3.2. Transmission of ARGs in Pipe Wall Biofilms

After the water treated by the water purification plant enters the water-supply network, the incomplete removal of ARB and ARGs spreads in the pipe network during long-distance transportation. In particular, biofilms on pipe walls provide favourable conditions for the spread of ARGs. Biofilms have a high bacterial density and relatively short bacterial spacing, which greatly increases the possibility of transmitting genetic material via HGT between bacteria. Engemann et al. found that tetracycline resistance genes (*tetO*, *tetW*, *tetM*, *tetQ*, *tetB*) can be integrated into biofilms, indicating that biofilms may serve as long-term repositories for ARGs [115]. Siedlecka et al. investigated the presence of ARB and ARGs in water-supply networks in Poland. Bacteria resistant to ceftazidime, ampicillin, ciprofloxacin, and tetracycline have been detected, and ARGs such as *blaTEM*, *ampC*, and *sulI* have also been identified [116]. Additionally, studies have shown that disinfection processes in water treatment plants can alter microbial community composition, thereby increasing the relative abundance of ARGs. The presence of biofilms in the water supply network, along with the co-selection effects of disinfectants [117] and heavy metals [118,119], may further promote the spread of ARGs.

**Figure 6:**
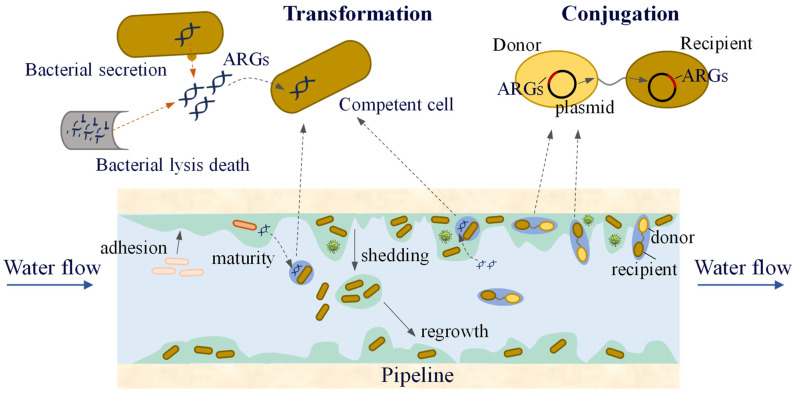
The transfer of ARGs in a water distribution system.

Conjugation and transformation are the two most common modes of HGT in water-supply network biofilms and water (Figure 6). Research has found [120] that low concentrations of chlorine and chloramine (0.05 and 0.1 mg/L) promote the conjugative transfer of ARGs within biofilms and water, suggesting that lower residual disinfectant levels in the water supply network may increase the risk of ARG transmission. At the same time, low concentrations of carbon sources can also increase the frequency of ARG conjugative transfer. The EPS in biofilms can be used as an energy source for nutrient deprivation. Therefore, compared to water, the increase in the frequency of ARG conjugative transfer in biofilms under carbon source limitation was smaller. The presence of ARBs and ARGs in the biofilm not only transfers more antibiotic resistance to the environment but also significantly improves the likelihood of pathogenic microorganisms acquiring resistance.

Both biofilm formation and shedding occurred in the pipeline. Biofilm development is a process in which microbial attachment and detachment reach a dynamic balance and continuous cycle. Shedding of the biofilm in the pipeline releases abundant microorganisms that are harmful to human health into the water, thus posing potential health risks for the use of reclaimed water. However, research on the patterns of biofilm formation in pipelines, as well as the composition of ARGs and microbial communities within biofilms, remains relatively scarce. Considering the unique environmental conditions of the pipeline network (such as the presence of disinfectants, nutrient-poor environments, and hydraulic shear forces), the transmission mechanisms of ARGs in biofilms and water, as well as the transfer patterns of ARGs and ARBs between biofilms and water, require further analysis.

## 4. Phage-Mediated ARGs in Biofilms

### 4.1. The Action of the Phages on Biofilms

Phages are viruses that infect bacteria and self-replicate. They are ubiquitous in nature and considered some of the most abundant species in the world, with approximately 10^31^, possibly exceeding the sum of all other species [121]. The phage life cycle can be divided into four types: lytic (virulent phages), lysogenic (temperate phages), chronic infection, and pseudolysogeny. Lytic phages specifically infect bacteria through a series of processes including attachment, injection, replication, assembly, lysis, and release. They replicate their genomes and proliferate within a short period, ultimately causing the lysis and death of the host bacterium. This type of phage is considered suitable for treating bacterial infections. Compared with traditional antimicrobial agents, such as antibiotics and disinfectants, phage therapy for bacterial infections has several advantages, including high specificity, better safety, stringent bactericidal activity, therapeutic effects with a single dose or lower, and a lower likelihood of inducing resistance [122,123].

The mechanism of phage resistance in bacterial biofilms involves diffusion via water channels, enzymatic degradation, infection of persister cells, and adsorption of bacterial flagella to promote phage infection of the host bacteria in the biofilm. (1) Bacterial biofilms are highly hydrated structures formed by voids (also known as water channels), which facilitate the diffusion of nutrients in bacterial biofilms and enable phages to penetrate bacterial biofilms through these water channels under the action of gravity [124]. Compared with traditional antibiotics acting on bacterial biofilms, phages do not consume high concentrations during the infiltration process. Instead, they can increase the number of phages through their active replication ability, causing microorganisms inside the bacterial biofilm to disintegrate, thereby interfering with the overall three-dimensional structure of the biofilm. (2) The phage genome encodes a variety of specific enzymes, such as endolysin/lysin, depolymerase, and virion-associated peptidoglycan hydrolases (VAPGHs), which can degrade bacterial peptidoglycans, capsular polysaccharides, and other substances to assist phages in quickly destroying the integrity of bacterial biofilms. This is one of the most important mechanisms through which phages act on bacterial biofilms. Lysin, a hydrolase produced at the end of the phage-infection cycle, specifically degrades peptidoglycans in the bacterial cell walls. Depolymerases, typically present in the tail fibres of phages, in the form of a tail spike protein (TSP) or free enzymes to promote bacterial cells disaggregation in biofilms and phage adsorption, can degrade bacterial surface structures such as capsular polysaccharides, lipopolysaccharides (LPS), O-antigen polysaccharide chains, or extracellular polysaccharides and even destroy peptides or lipids on the cell wall [125]. (3) Persister cells are metabolically inactive bacterial cells that are deep in the bacterial biofilm, showing very low cell growth and cell division rates. Antibiotics primarily target the metabolically active bacterial cells. However, when traditional antibiotics are used to treat bacterial biofilms, persister cells within the biofilm can enter a dormant state, making them nearly insensitive to antibiotics. Once treatment is stopped, persister cells can be reactivated as the host bacterium of the new bacterial biofilm forms, leading to bacterial infections and chronic infection recurrence [126]. Unlike antibiotics, lytic phages can efficiently infect and kill persister cells in a dormant state. (4) Phages can be adsorbed onto the flagella of motile bacteria to enhance their motility. Based on this feature, phages can ride on motile non-host bacteria (carriers), thus promoting phage infection of host bacteria within biofilms. Yu et al. [127] demonstrated that the lytic phage PHH01 could adsorb to the flagella of *Bacillus cereus*, effectively hitching a ride on the *B. cereus* carrier, thus making PHH01 not only 5.15 times more migratory than PHH01 alone but also 4.36 times more efficiently infected with the biofilm host bacteria *E. coli* than PHH01 alone. This study confirmed that the behaviour of phages could enhance their ability to infect bacteria within biofilms.

The relationship between phages and bacteria within biofilms is not a simple predator-prey dynamic but involves complex interactions. Phages have effective anti-biofilm effects through mechanisms such as encoding specific degradative enzymes and can also promote the formation of bacterial biofilms in some cases, such as low concentrations of phages, specific phages, or prophages. (1) Low phage concentrations stimulate biofilm formation. The researchers treated the biofilm of *E. coli* K-12 with polyvalent lytic phages at low concentrations (10^2^–10^4^ phages/mL) for 6 h. The results showed that compared with the untreated group, the expression of QS genes, polysaccharide production genes, and curli synthesis genes in *E. coli* K-12 biofilm increased. This leads to a higher polysaccharide and extracellular DNA (eDNA) content in the biofilm matrix and promotes biofilm formation [128]. (2) Specific phages contribute to bacterial biofilm formation. Because mild, non-enveloped filoviruses can infect *P. aeruginosa*, Pf phages can contribute to *P. aeruginosa* biofilm formation. Studies have found that the upregulation of Pf phage genes is a common feature of biofilm formation in different laboratory settings and that *P. aeruginosa* biofilms grown under different conditions are associated with the production of a large number of overlapping infected Pf phages [129]. (3) The prophages release matrix components required for bacterial biofilm assembly. A linear plasmid-like prophage, xhp1, contributes to the formation of XH001 biofilms by spontaneously inducing and releasing eDNA, providing the matrix components required for *Actinomyces odontolyticus* XH001 biofilm assembly. In conclusion, interactions between phages and bacterial biofilms are relatively complex. Further in-depth understanding and research are required to develop efficient strategies for future anti-bacterial biofilms.

### 4.2. Phage-Mediated Transfer of ARGs

The process by which phages initially acquire ARGs typically occurs through transduction. Specifically, this process can occur through three pathways: the accidental acquisition of bacterial genes, gene exchange between bacteria, and HGT. Most mobile resistance genes are located on plasmids and their associated genetic elements and undergo gene transfer via direct contact with bacteria [130]. Moreover, a large amount of genetic information on bacterial genomes is derived from phages. Each bacterial strain detects 2.6 prophages, and many bacterial genomes contain 3–10% prophage DNA, which may carry a large number of important functional groups [131,132]. As vectors for gene transfer, phages can facilitate the spread of ARGs within bacterial populations. Studies have found that phages carrying resistance genes can transfer these genes into bacteria, leading to changes in bacterial resistance [133]. Therefore, phages play an important role in altering the genetic composition of bacteria and enhancing their resistance, virulence, and adaptability.

Although phage genomes are relatively small (a few to hundreds of kb), they can transfer genes from different host bacteria via transduction, resulting in high genome diversity and complex evolutionary relationships [134]. Shousha et al. [135] isolated 243 phages from chicken meat, 24.7% of which could make *E. coli* resistant to antibiotics (ampicillin, tetracycline, chloramphenicol, β-lactamase) through transduction. Haaber et al. [136] also found that phages released from *S. aureus* allowed their hosts to acquire streptomycin-resistance genes from neighbouring cells. With the continuous development of research methods and technologies, an increasing number of viral fragments carrying resistance genes have been identified [137,138,139]. However, the sample size in laboratory studies is relatively small, making it difficult to comprehensively represent the diversity of phages in nature. This may lead to an underestimation of the situation where phages carry ARGs, as there may be significant differences in the genetic composition and functions of phages in different environments. This further confirms the important role of phages in HGT and gene recombination [140].

From the perspective of the association between phages and bacterial virulence, Karaolis et al. [141] first proposed the concept of a virulence island and identified a Vibrio pathogenicity island (VPI) present in pathogenic strains but absent in non-pathogenic strains. The VPI is 39.5 kb, with a relatively low GC content (approximately 35%), contains integrase and transposase genes, and has an att site on each side. It is located near the 10Sa RNA gene (ssrA). These features, which are similar to those of phages, suggest that virulence islands may be derived from phages. Faruque et al. [142] found that certain phages can act as cofactors to promote HGT. Santoriello et al. [143] found that phages enable bacteria to acquire special gene clusters through prophage transfer, which helps them acquire virulence genes and enhances their adaptability to the environment. It can be seen that phage-mediated gene transfer can help bacteria acquire virulence-related genes, thereby enhancing their virulence. This poses a significant threat to public health and should be given attention.

**Figure 7:**
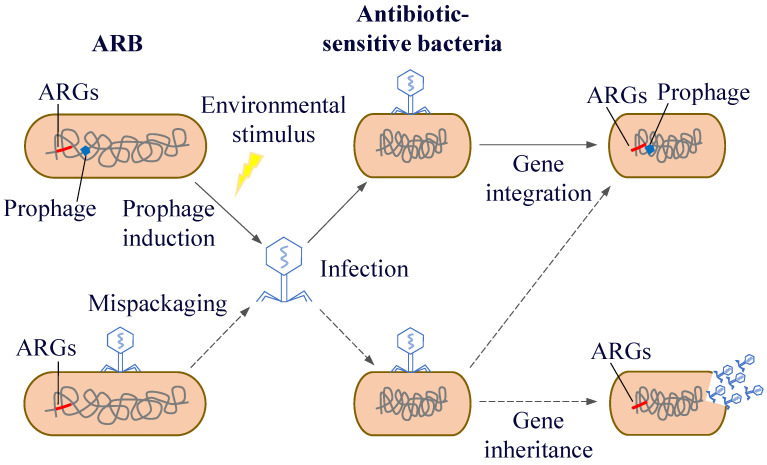
Horizontal transfer of resistance genes mediated by phages.

After phages infect bacteria, there are two life cycles: the lysogenic and the lytic life cycle. During the process of lysogenic conversion, ARGs are generally not expressed immediately because the phage is in a latent state. However, these genes will be passed on to the descendant bacteria as part of the bacterial genetic material. And under specific circumstances, the phage will shift from the lysogenic state to the lytic cycle, at which point the resistance genes will be expressed and spread. Driven by different mechanisms, phages can act as carriers for the spread of resistance genes through generalised and localised transduction, thus transferring their ARGs from one host to another [144]. Generalised transduction can randomly transduce any gene into the host bacterium, including ARGs. Fillol-Salom et al. [145] found that generalised transduction is beneficial for both phages and host bacteria. By integrating temperate phages, bacteria can carry ARGs that promote bacterial survival and phage proliferation. Interactions between phages and their hosts facilitate better survival in rapidly changing environments. In localised transduction, when prophage gene fragments are detached from the host chromosome, host genes near their integration location are mistakenly excised and incorporated into the progeny phage genome. Subsequently, these genes spread among the host bacteria through HGT (Figure 7). Localised transduction contributes less to gene transfer than generalised transduction; however, the positional specificity of localised transduction imparts a special function to localised transduction phages. Localised transduction can be used to construct deletion mutants in most mycobacteria [146]. Tufariello et al. [147] found that phage-mediated localised transduction increased the efficiency of constructing knockout strains by more than 100-fold. This significantly aids the creation of more diverse gene knockout libraries, enables faster acquisition of deletion mutants, and facilitates the development of antimicrobial agents and vaccines. In localised transduction, prophages enhance bacterial environmental fitness or virulence by secreting phage-encoded toxins, regulating bacterial biofilms, mediating bacterial infections, and controlling bacterial cell activity. The cell wall surface protein CwpV encoded by *Clostridium difficile* prophage ΦCD38-2 can increase toxin expression in the bacterial lysogenic cycle and induce the expression of other bacterial genes associated with carbon metabolism [148]. Moreover, phages can spread between different environmental mediators and human activities, which in turn influence the microbial community structure and distribution of ARGs.

In terms of the host range and transmission characteristics of gene transfer, phage-mediated gene transfer occurs across host cells, and the gene donor and recipient bacteria do not need to be simultaneously present in the same biological community at the same time. Some phages can infect different hosts across species of bacterial taxonomic genera, orders, or even at the phylum level [149]. For phages with a wide host range, transduction may also be across consortia. Phages are protected by a protein coat and can survive longer than other gene carriers. This may delay the transmission of genetic information and extend the timescale of its effects [150]. In addition, prophages are hidden in host bacteria and only lyse the host under certain conditions; therefore, phage-mediated resistance gene transmission is more difficult to detect and control. Therefore, studying the ARGs carried by phages and phage-mediated HGT in different environments, as well as exploring the relationship between phages, host bacteria, and resistance genes, is of great significance for mitigating environmental and human health risks. Ross et al. [151] conducted a transduction experiment using soil phages to infect *E. coli* K-12. Target genes were detected in all phage DNA samples, and the addition of antibiotics (ampicillin, cefotaxime, and sulfamethoxazole) promoted phage-mediated horizontal transfer of ARGs in soil. Fard et al. [152] designed interbacterial transduction experiments and showed that antibiotic resistance could be transferred between strains under phage-mediated conditions.

It is worth noting that HGT occurs more rapidly in biofilms than in planktonic cells. Within biofilms, the evolution of bacteria and the development of resistant bacteria can be achieved by the transfer of MGEs such as plasmids that encode ARGs. This gene transfer can be facilitated by exposing biofilms to sub-minimum inhibitory concentrations (sub-MICs) of antibiotics. Therefore, biofilms are important reservoirs for the spread of AMR [153,154]. Studies have established a positive correlation between biofilm formation and AMR in *Acinetobacter baumannii*, with extensive drug-resistant (XDR) strains forming more robust biofilms than multi-drug-resistant (MDR) strains [155,156,157]. However, other reports have suggested that XDR strains tend to form weaker biofilms than non-MDR and MDR strains [158,159]. This suggests an urgent need for a better understanding of the regulatory mechanisms underlying biofilm formation and AMR.

In current laboratory studies on phage-mediated gene transfer, model strains such as *E. coli* and *S. aureus* are commonly used. While these strains have well-defined genetic backgrounds and are easy to culture, they do not fully represent the diverse bacteria found in natural environments, which exhibit complex genetic traits and physiological characteristics. Bacteria in natural settings may have unique defence mechanisms against phage infections and gene transfer, which may be absent in model strains. As a result, experimental models based on these strains may either overestimate or underestimate the impact of phages on bacterial gene transfer in real-world environments, leading to findings that do not accurately reflect the actual dynamics of phage-mediated ARG transmission in nature. Additionally, in natural environments, bacteria and phages are exposed to a mixture of various antibiotics, with antibiotic concentrations fluctuating over time and across different locations, which greatly differ from the controlled conditions in laboratory experiments. This variability makes it difficult to predict the dynamic process of phage-mediated ARG transmission in complex environments. Therefore, it is crucial to conduct studies in more realistic settings. For instance, in soil environments, aside from phages and target bacteria, a wide array of other microorganisms can indirectly influence phage-mediated gene transfer through mechanisms like the production of antibacterial substances or nutrient competition [160]. Only by researching real-world environments can we fully understand the impact of these complex ecological interactions on ARG transmission and more accurately assess the risks associated with AMR spread. 

The above key points have been summarized in Table 2.

## 5. The Impact of ARG Transfer on Public Health and the Corresponding Response Strategies

### 5.1. The Spread of ARGs and Its Threat to Public Health

The transfer process of ARGs has had a profound impact on public health and poses challenges to the formulation of AMR control policies and mitigation strategies. ARGs spread widely through gene transfer mechanisms between bacteria, especially through HGT, phage-mediated gene transfer, and the resistance gene pool in the environment [161,162,163]. These transfer pathways enable resistance genes to spread rapidly within bacterial populations, leading to an increasing number of pathogenic bacteria developing drug resistance. The spread of resistance genes is not limited to the same species of bacteria but can also occur across different species or groups, further exacerbating the diversity and complexity of drug-resistant bacteria.

With the widespread dissemination of ARGs, an increasing number of common infectious pathogens have developed resistance to antibiotics, posing a serious threat to public health. Traditional antibiotic treatment methods are gradually becoming ineffective, making clinical treatment increasingly difficult. For instance, the complications and mortality rates caused by infections have significantly increased. Especially among patients with weakened immune systems, drug-resistant infections often lead to severe consequences [164]. At the same time, the emergence of drug-resistant bacteria has also resulted in an increase in treatment costs and an extension of hospital stays, imposing a huge burden on the public health system. The formation of drug-resistant bacteria, particularly the emergence of MDR bacteria and superbugs, has further exacerbated this problem. MDR bacteria are resistant to a variety of commonly used antibiotics, leaving extremely limited treatment options. In some cases, superbugs may even be resistant to almost all existing antibiotics, leading to some infections becoming untreatable.

### 5.2. Global Cooperation and Control Strategies to Address the Antibiotic Resistance Crisis

The impact of the transfer process of ARGs on AMR control policies is mainly reflected in the following aspects. Firstly, the widespread dissemination of resistance genes calls for strengthened global management of antibiotic use. Whether in the fields of hospitals, agriculture, or public health, the excessive or inappropriate use of antibiotics will accelerate the selective pressure of resistance genes. In order to effectively reduce the emergence of drug-resistant bacteria, the rational use of antibiotics and the restriction of unnecessary antibiotic use have become key strategies. In the medical environment, especially when treating minor infections, the abuse of broad-spectrum antibiotics must be avoided to prevent the accumulation and spread of resistance genes.

Secondly, infection control measures in hospitals and public health settings need to be further strengthened. By improving disinfection and isolation measures in hospitals, the proliferation of ARB within wards can be effectively controlled. Additionally, closely monitoring and controlling antibiotic use in hospital settings, as well as reducing the occurrence of resistant bacteria through early diagnosis and precise treatment, are also effective control strategies.

Thirdly, it is particularly urgent to promote the development of new antibiotics and alternative treatment methods. Currently, many commonly used antibiotics are facing the threat of resistance. Developing new antibiotics or alternative treatments, such as phage therapy and immunotherapy, that can effectively combat resistant bacteria will be crucial in addressing this crisis. These new treatment approaches offer hope for treating difficult-to-cure MDR infections and can effectively fill the treatment gap caused by antibiotic resistance.

Finally, the transfer of ARGs is a global issue that requires cooperation and information sharing between countries. Through global coordination and joint efforts, sharing data on antibiotic use, resistance monitoring, and research and development outcomes can better address the resistance crisis. International cooperation can also help countries strengthen local drug management policies and establish cross-border standards to further curb the spread of resistance genes.

Overall, the impact of ARG transfer on public health is profound and complex. This issue not only affects the healthcare systems of individual countries but also concerns global health security. To effectively control AMR, comprehensive measures are needed, including rational use of antibiotics, strengthening infection control, promoting new drug development, and enhancing international cooperation. These strategies will help slow the spread of resistance genes and, in turn, protect public health.

## 6. Conclusions

ARGs are emerging pollutants that are attracting increasing attention. Unlike other pollutants, ARGs in the environment can be transferred horizontally and spread to pathogens, making the environment a natural reservoir of ARGs. The occurrence characteristics of ARGs in various environmental media have always been a key focus of ARG research. Compared with ARGs in soil, water, and atmospheric environments, there is relatively little research on biofilms. However, as a “shelter” for bacteria, biofilms do not lack resistant bacteria and resistance genes, and HGT occurs more frequently in biofilms than between free-floating cells. Further research reveals that ARGs and their HGT phenomena are widely present in microplastics, activated sludge, and pipe-wall biofilms.

Similarly, MPs, as emerging pollutants, accumulate in the environment, owing to their small size and non-degradability, and easily adsorb harmful substances, including a large number of pathogenic microorganisms. Moreover, because MP biofilms provide an excellent habitat for bacteria, the diversity and abundance of ARGs are also higher than those of the surrounding environment. Currently, research on MPs is a popular topic, mainly focusing on the migration of MPs in aquatic environments and their impact on bacterial communities and diversity, concentrating on the hazards of MPs to the environment and pollution control. However, studies combining MPs with ARGs are relatively scarce, and even fewer have been conducted on ARGs in MP biofilms. Given that MPs and ARGs migrate and are difficult to remove from the environment, their combination is undoubtedly a major threat to environmental health and safety. Therefore, strengthening the study of bacterial communities, ARGs, and their interactions in MP biofilms may provide a breakthrough for the treatment of these two emerging pollutants.

Sludge is solid waste produced during sewage treatment. It contains pollutants removed from sewage, such as organic matter, inorganic matter, and microorganisms (including those that carry ARGs). Although treatment systems have reduced the concentration of ARGs in effluent, a significant amount of ARGs is still retained in the sludge. In addition to focusing on the quality of the effluent, it is also important to focus on the treatment and disposal methods of sludge to minimise the potential environmental risks posed by ARGs. As a unique biofilm, AGS contains a variety of microorganisms, and the granulation of AGS is the process of biofilm formation. The selection pressure for antibiotics stimulates this process. At present, it has been shown that QS has a certain influence on particle formation and stability, and QS affects the spread of ARGs. However, no systematic analysis has been conducted on the fate of ARGs in AGS formation under environmentally related concentrations of antibiotics.

Most microorganisms in the water supply pipeline exist in biofilms on pipe walls, which are more likely to survive in the water supply network, making the biofilm on the pipe wall a reservoir of genetic material. Unlike other biofilms, the biofilm on the pipe wall is affected by the water supply system, such as the temperature, hydrological conditions, operating parameters, and pipe materials. Moreover, the biofilm has a shedding process that releases large amounts of microorganisms that are harmful to human health into the water. Conjugation and transformation are the two main mechanisms of ARG transfer in pipe wall biofilms. The complex environment in a pipe affects this process. For example, the presence of disinfectants promotes the conjugation transfer of ARGs in biofilms and water. In addition, the detachment of biofilms caused by hydraulic erosion brings ARGs into the water, which increases the difficulty of studying the horizontal transfer of ARGs.

Phages are now used in clinical biological treatments for diseases caused by drug-resistant bacteria, especially in phage cocktail therapies. Moreover, there has been extensive research on the use of phages against drug-resistant bacteria and bacterial biofilms in the environment. Phages can not only resist biofilms but also interact with them and promote biofilm formation under certain conditions. In addition, phages, which are important mediators of gene transfer, can alter the genetic composition of bacteria and enhance their drug resistance, virulence, and adaptability. Phage-mediated transduction is ubiquitous in the environment. The effects caused by phage transduction are long in time span and difficult to control. Research on phages against drug-resistant bacterial biofilms and phage-mediated ARG transfer in the environment has made some progress, but there is a lack of research on phage transduction in the biofilm environment. The relationship between drug resistance and biofilm formation and its coregulatory mechanisms still needs to be further explored. Large-scale studies should be carried out in the future to monitor the transfer of ARGs in environmental and clinical biofilms and to analyse the mechanisms of phages’ involvement in this process.

## Figures and Tables

**Table 1:** Nanomaterials and their relationship with ARGs.

Nanomaterials	The Relation with ARGs
Cu nanocomposites	Reduced the abundance of ARGs [62].
Nano-Fe_2_O_3_	Increased the transfer of the RP4 plasmid from *E. coli* to aquatic bacterial communities [65].
Nano-Al_2_O_3_	Increased the inter-genus and intra-genus transfer efficiency [66].
Nano-ZnO	Increased the conjugation and horizontal transfer efficiency [69].
Nano-CnO	Promoted the environmental transmission of ARGs [71].
GO	Enhanced the ARGs’ transfer efficiency [68]. In the actual environment, GO can form complexes with extracellular antibiotics, inhibit the uptake of antibiotics by bacteria, reduce the abundance of ARGs, and also decrease the transfer efficiency of integrons carrying ARGs under optimal conditions [70].

**Table 2:** Phage-mediated transfer of ARGs.

Topic	Key Points	References
The pathways through which phages acquire ARGs	Through transduction, including the accidental acquisition of bacterial genes, gene exchange between bacteria, and HGT; most mobile resistance genes are located on plasmids and related genetic elements, and the genes are transferred through direct contact with bacteria.	[130]
The relationship between phages and bacterial genomes	A large amount of genetic information in the bacterial genome is derived from phages. Each bacterial strain has 2.6 prophages detected, and many bacterial genomes contain 3–10% prophage DNA, which may carry a large number of important functional groups.	[131,132]
The relationship between phages and the virulence of bacteria	The concept of the virulence island was proposed. It has characteristics similar to those of phages and may be derived from phages. Certain phages can act as cofactors to promote HGT, help bacteria acquire virulence gene clusters through prophage transfer, and enhance their virulence.	[141,142,143]
The life cycle of phages and gene transfer	There are two life cycles: the lysogenic and the lytic cycle. Resistance genes are spread through generalised transduction (which can randomly transduce any gene, including ARGs, and is beneficial to both phages and host bacteria) and localised transduction (when prophage fragments are detached from the host chromosome, they mistakenly excise the nearby host genes and integrate them into the genome of the progeny phages, playing an important role in constructing deletion mutants in most mycobacteria).	[144,145,146,147]
Characteristics of phage-mediated ARG transfer	It can cross host cells, and the gene donor and recipient bacteria do not need to be in the same biological community simultaneously. Some phages have a wide host range and can infect across different bacterial taxonomic genera, orders, and even phyla. Protected by a protein coat, phages can survive for a long time. Prophages are hidden within host bacteria, and the spread of resistance genes mediated by them is difficult to detect and control.	[149,150]
The relationship between biofilms, HGT, and antibiotic resistance	HGT occurs more rapidly in biofilms than in planktonic cells. The evolution of bacteria and the emergence of drug-resistant bacteria in biofilms can be achieved through the transfer of MGEs (such as plasmids) encoding ARGs, and exposure to antibiotics at sub-MICs can promote this process. There are different views on the relationship between biofilm formation of *A. baumannii* and AMR, and it is necessary to further clarify the regulatory mechanism.	[153,154,155,156,157,158,159]
Limitations of laboratory studies	Model strains such as *E. coli* and *S. aureus* are often selected, which cannot fully represent the bacteria in the natural environment. Model strains may lack the unique defence mechanisms that bacteria in the natural environment possess. This may lead to the experimental models overestimating or underestimating the impact of phages on bacterial gene transfer. In the natural environment, bacteria and phages are faced with the mixed pollution of multiple antibiotics, and the concentrations of these antibiotics change over time and space. This is greatly different from the laboratory conditions, making it difficult to predict the dynamic process of phage-mediated spread of ARGs in complex environments.	

## Data Availability

The original contributions presented in this study are included in the article/Supplementary Materials. Further inquiries can be directed to the corresponding author.

## Supplementary Materials

The following supporting information can be downloaded at https://www.mdpi.com/article/10.3390/microorganisms13050997/s1: Table S1: Classification of the ARGs.

## References

[1] Willyard C. (2017). The drug-resistant bacteria that pose the greatest health threats. Nature.

[2] Forsberg K.J., Reyes A., Wang B., Selleck E.M., Sommer M.O.A., Dantas G. (2012). The shared antibiotic resistome of soil bacteria and human pathogens. Science.

[3] Huijbers P.M.C., Flach C.F., Larsson D.G.J. (2019). A conceptual framework for the environmental surveillance of antibiotics and antibiotic resistance. Environ. Int..

[4] Pruden A., Pei R., Storteboom H., Carlson K.H. (2006). Antibiotic resistance genes as emerging contaminants: Studies in northern Colorado. Environ. Sci. Technol..

[5] Costa V.M., King C.E., Kalan L., Morar M., Sung W.W.L., Schwarz C., Froese D., Zazula G., Calmels F., Debruyne R. (2011). Antibiotic resistance is ancient. Nature.

[6] Arias-Andres M., Klümper U., Rojas-Jimenez K., Grossart H.P. (2018). Microplastic pollution increases gene exchange in aquatic ecosystems. Environ. Pollut..

[7] Su J.Q., Huang F.Y., Zhu Y.G. (2013). Antibiotic resistance genes in the environment. Biodivers. Sci..

[8] Wang J.Y., An X.L., Zhang H.M., Su J.Q. (2024). Manure application enriches phage-associated antimicrobial resistance and reconstructs ecological network of phage-bacteria in paddy soil. Soil Biol. Biochem..

[9] Aziz R., Colomer-Lluch M., Jofre J., Munisa M. (2011). Antibiotic resistance genes in the bacteriophage DNA fraction of environmental samples. PLoS ONE.

[10] Marti E., Variatza E., Balcazar J.L. (2014). Bacteriophages as a reservoir of extended-spectrum beta-lactamase and fluoroquinolone resistance genes in the environment. Clin. Microbiol. Infect..

[11] Calero-Caceres W., Melgarejo A., Colomer-Lluch M., Stoll C., Lucena F., Jofre J., Muniesa M. (2014). Sludge as a potential important source of antibiotic resistance genes in both the bacterial and bacteriophage fractions. Environ. Sci. Technol..

[12] Wang L., Luo Y., Mao D., Zhou Q. (2010). Transport of antibiotic resistance genes in environment and detection methods of antibiotic resistance. Chin. J. Appl. Ecol..

[13] Zhu Y.G., Johnson T.A., Su J.-Q., Tiedje J.M. (2013). Diverse and abundant antibiotic resistance genes in Chinese swine farms. Proc. Natl. Acad. Sci. USA.

[14] Brown M.G., Mitchell E.H., Balkwill D.L. (2008). Tet 42, a novel tetracycline resistance determinant isolated from deep terrestrial subsurface bacteria. Antimicrob. Agents Chemother..

[15] Huang F.Y., Zhou S.Y.D., Wang J.N., Su J.Q., Li H. (2021). Profiling of antibiotic resistance genes in different croplands. Environ. Sci..

[16] Aarestrup F.M., Agerso Y., Gerner-Smidt P., Madsen M., Jensen L.B. (2000). Comparison of antimicrobial resistance phenotypes and resistance genes in *Enterococcus faecalis* and *Enterococcus faecium* from humans in the community, broilers, and pigs in Denmark. Diagn. Microbiol. Infect. Dis..

[17] Szekeres E., Chiriac C.M., Baricz A., Szőke-Nagy T., Lung I., Soran M.-L., Rudi K., Dragos N., Coman C. (2018). Investigating antibiotics, antibiotic resistance genes, and microbial contaminants in groundwater in relation to the proximity of urban areas. Environ. Pollut..

[18] Knapp C.W., Zhang W., Sturm B.S.M., Graham D.W. (2010). Differential fate of erythromycin and beta-lactam resistance genes from swine lagoon waste under different aquatic conditions. Environ. Pollut..

[19] Zhu Y.G., Zhao Y.I., Li B., Huang C.-L., Zhang S.-Y., Yu S., Chen Y.-S., Zhang T., Gillings M.R., Su J.-Q. (2017). Continental-scale pollution of estuaries with antibiotic resistance genes. Nat. Microbiol..

[20] Suzuki S., Ogo M., Miller T.W., Shimizu A., Takada H., Siringan M.A.T. (2013). Who possesses drug resistance genes in the aquatic environment? sulfamethoxazole (SMX) resistance genes among the bacterial community in water environment of Metro-Manila, pHilippines. Front. Microbiol..

[21] Toth M., Smith C., Frase H., Mobashety S., Vakulenko S. (2010). An antibiotic-resistance enzyme from a deep-sea bacterium. J. Am. Chem. Soc..

[22] Su H.C., Pan C.G., Ying G.G., Zhao J.-L., Zhou L.-J., Liu Y.-S., Tao R., Zhang R.-Q., He L.-Y. (2014). Contamination profiles of antibiotic resistance genes in the sediments at a catchment scale. Sci. Total Environ..

[23] Yang Y., Liu W., Xu C., Wei B., Wang J. (2017). Antibiotic resistance genes in lakes from middle and lower reaches of the Yangtze River, China: Effect of land use and sediment characteristics. Chemosphere.

[24] Dang C., Xia Y., Zheng M., Liu T., Liu W., Chen Q., Ni J. (2020). Metagenomic insights into the profile of antibiotic resistomes ina large drinking water reservoir. Environ. Int..

[25] Zhang K., Xin R., Li K.J., Wang Q., Wang Y.-N., Xu Z.-H., Cui X.-C., Wei W. (2021). Seasonal variation and influencing factor analysis of antibiotic resistance genes in water supply reservoirs of gentral China. Environ. Sci..

[26] Nnadozie C.F., Odume O.N. (2019). Freshwater environments as reservoirs of antibiotic resistant bacteria and their role in the dissemination of antibiotic resistance genes. Environ. Pollut..

[27] Madsen A.M., Nielsen U., Uhrbrand K., Schultz A.C., Koivisto A.J. (2017). Assessment of airborne bacteria and noroviruses in air emission from a new highly-advanced hospital wastewater treatment plant. Water Res. J. Int. Water Assoc..

[28] Li J., Zhou L., Zhang X., Xu C., Dong L., Yao M. (2016). Bioaerosol emissions and detection of airborne antibiotic resistance genes from a wastewater treatment plant. Atmos. Environ..

[29] Wang Y., Wang C., Song L. (2019). Distribution of antibiotic resistance genes and bacteria from six atmospheric environments: Exposure risk to human. Sci. Total Environ..

[30] Gao M., Jia R., Qiu T., Han M., Wang X. (2017). Size-related bacterial diversity and tetracycline resistance gene abundance in the air of concentrated poultry feeding operations. Environ. Pollut..

[31] Letourneau V., Masse D., Duchaine C., Nehmé B., Mériaux A., Cormier Y. (2010). Human pathogens and tetracycline-resistant bacteria in bioaerosols of swine confinement buildings and in nasal flora of hog producers. Int. J. Hyg. Environ. Health.

[32] Gilbert Y., Veillette M., Duchaine C. (2010). Airborne bacteria and antibiotic resistance genes in hospital rooms. Aerobiologia.

[33] Shiomori T., Miyamoto H., Makishima K. (2001). Significance of airborne transmission of methicillin-resistant *Staphylococcus aureus* in an otolaryngology-head and neck surgery unit. Arch. Otolaryngol. Head Neck Surg..

[34] Wang H., Hou L., Liu Y., Liu K., Zhang L., Huang F., Wang L., Rashid I., Hu A., Yu C. (2021). Horizontal and vertical gene transfer drive sediment antibiotic resistome in an urban lagoon system. J. Environ. Sci..

[35] Lerminiaux N.A., Cameron A.D.S. (2019). Horizontal transfer of antibiotic resistance genes in clinical environments. Can. J. Microbiol..

[36] Pazda M., Kumirska J., Stepnowski P., Mulkiewicz E. (2019). Antibiotic resistance genes identified in wastewater treatment plant systems—A review. Sci. Total Environ..

[37] Balcazar J.L. (2014). Bacteriophages as vehicles for antibiotic resistance genes in the environment. PLoS Pathog..

[38] Kucho K., Kakoi K., Yamaura M., Iwashita M., Abe M., Uchiumi T. (2013). Codon-optimized antibiotic resistance gene improves efficiency of transient transformation in Frankia. J. Biosci..

[39] Shakibaie M.R., Jalilzadeh K.A., Yamakanamardi S.M. (2009). Horizontal transfer of antibiotic resistance genes among gram negative bacteria in sewage and lake water and influence of some physico-chemical parameters of water on conjugation process. J. Environ. Biol..

[40] Katale B.Z., Misinzo G., Mshana S.E., Chiyangi H., Campino S., Clark T.G., Good L., Rweyemamu M.M., Matee M.I. (2020). Genetic diversity and risk factors for the transmission of antimicrobial resistance across human, animals and environmental compartments in East Africa: A review. Antimicrob. Resist. Infect. Control.

[41] Letten A.D., Hall A.R., Levine J.M. (2021). Using ecological coexistence theory to understand antibiotic resistance and microbial competition. Nat. Ecol. Evol. Nat. Publ. Group.

[42] Rodriguez-Mozaz S., Chamorro S., Marti E., Huerta B., Gros M., Sànchez-Melsió A., Borrego C.M., Barceló D., Balcázar J.L. (2015). Occurrence of antibiotics and antibiotic resistance genes in hospital and urban wastewaters and their impact on the receiving river. Water Res..

[43] Luo Y., Mao D., Rysz M., Zhou Q., Zhang H., Xu L., Alvarez P.J.J. (2010). Trends in antibiotic resistance genes occurrence in the Haihe river, China. Environ. Sci. Technol. Am. Chem. Soc..

[44] Graham D.W., Olivares-Rieumont S., Knapp C.W., Lima L., Werner D., Bowen E. (2011). Antibiotic resistance gene abundances associated with waste discharges to the Almendares River near Havana, Cuba. Environ. Sci. Technol..

[45] Larsson D.G.J., Cecilia D.P., Nicklas P. (2007). Effluent from drug manufactures contains extremely high levels of pharmaceuticals. J. Hazard. Mater..

[46] Kristiansson E., Fick J., Janzon A., Grabic R., Rutgersson C., Weijdegård B., Söderström H., Larsson D.G.J. (2011). Pyrosequencing of antibiotic-contaminated river sediments reveals high levels of resistance and gene transfer elements. PLoS ONE.

[47] Devirgiliis C., Zinno P., Stirpe M., Barile S., Perozzi G. (2014). Functional screening of antibiotic resistance genes from a representative metagenomic library of food fermenting microbiota. BioMed Res. Int..

[48] Graham D. (2015). Antibiotic resistance in the environment: Not the usual suspects. Chem. Biol..

[49] Liu Y.Y., Wang Y., Walsh T.R., Yi L.-X., Zhang R., Spencer J., Doi Y., Tian G., Dong B., Huang X. (2016). Emergence of plasmid-mediated colistin resistance mechanism MCR-1 in animals and human beings in China: A microbiological and molecular biological study. Lancet Infect. Dis..

[50] Wichmann F., Udikovic-Kolic N., Andrew S., Handelsman J. (2014). Diverse antibiotic resistance genes in dairy cow manure. mBio.

[51] Clemente J.C., Pehrsson E.C., Blaser M.J., Sandhu K., Gao Z., Wang B., Magris M., Hidalgo G., Contreras M., Noya-Alarcón Ó. (2015). The microbiome of uncontacted Amerindians. Sci. Adv..

[52] Gibson M.K. (2015). Improved annotation of antibiotic resistance determinants reveals microbial resistomes cluster by ecology. ISME J..

[53] Enne V., Bennett P.M., Livermore D.M., Hall L.M.C. (2004). Enhancement of host fitness by the 5uZ2-coding plasmid p9123 in the absence of selective pressure. J. Antimicrob. Chemother..

[54] Chen X., Du Z., Song X., Wang L., Wei Z., Jia L., Zhao R. (2023). Evaluating the occurrence frequency of horizontal gene transfer induced by different degrees of heavy metal stress. J. Clean. Prod..

[55] He X., Xu Y., Chen J., Ling J., Li Y., Huang L., Zhou X., Zheng L., Xie G. (2017). Evolution of corresponding resistance genes in the water of fish tanks with multiple stresses of antibiotics and heavy metals. Water Res..

[56] Xu S., Liu Y., Wang R., Zhang T., Lu W. (2022). Behaviors of antibiotic resistance genes (ARGs) and metal resistance genes (MRGs) during the pilot-scale biophysical drying treatment of sewage sludge: Reduction of ARGs and enrichment of MRGs. Sci. Total Environ..

[57] Li X., Xu Y., Ling J., Zhou J., He X., Huang L., Zheng L., Qiao Q. (2017). Impacts of cefotaxime sodium and heavy metals on AmpC β-lactamase resistance gene transfer. Acta Sci. Circumstantiae.

[58] Zhang Y., Gu A.Z., Cen T., Li X., He M., Li D., Chen J. (2018). Sub-inhibitory concentrations of heavy metals facilitate the horizontal transfer of plasmid-mediated antibiotic resistance genes in water environment. Environ. Pollut..

[59] Thomas J.C., Oladeinde A., Kieran T.J., Finger J.W., Bayona-Vásquez N.J., Cartee J.C., Beasley J.C., Seaman J.C., McArthur J.V., Rhodes O.E. (2020). Co-occurrence of antibiotic, biocide, and heavy metal resistance genes in bacteria from metal and radionuclide contaminated soils at the Savannah River Site. Microb. Biotechnol..

[60] Knapp C.W., Mccluskey S.M., Singh B.K., Campbell C.D., Hudson G., Grham D.W. (2011). Antibiotic resistance gene: Abundances correlate with metal and geochemical conditions in archived Scottish soils. PLoS ONE.

[61] Devarajan N., Laffite A., Graham N.D., Meijer M., Prabakar K., Mubedi J.I., Elongo V., Mpiana P.T., Ibelings B.W., Wildi W. (2015). Accumulation of clinically relevant antibiotic-resistance genes, bacterial load, and metals in freshwater lake sediments in Central Europe. Environ. Sci. Technol..

[62] Ji X., Tang Y., Ye J., Wu S., Hou M., Huang S., Wang R. (2022). The effect of carbon-basedcopper nanocomposites on Microcystis *aeruginosa* and the movability of antibiotic resistance genes in urban water. Chemosphere.

[63] Su Y., Wu D., Xia H., Zhang C., Shi J., Wilkinson K.J., Xie B. (2019). Metallic nanoparticles induced antibiotic resistance genes attenuation of leachate culturable microbiota: The combined roles of growth inhibition, ion dissolution and oxidative stress. Environ. Int..

[64] Huang Z., Zhao W., Xu T., Zheng B., Yin D. (2019). Occurrence and distribution of antibiotic resistance genes in the water and sediments of Qingcaosha Reservoir, Shanghai, China. Environ. Sci. Eur..

[65] Pu Q., Fan X.T., Sun A.Q., Pan T., Li H., Lassen S.B., An X.L., Su J.Q. (2021). Co-effect of cadmium and iron oxide nanoparticles on plasmid-mediated conjugative transfer of antibiotic resistance genes. Environ. Int..

[66] Qiu Z., Yu Y., Chen Z., Jin M., Yang D., Zhao Z., Wang J., Shen Z., Wang X., Qian D. (2012). Nanoalumina promotes the horizontal transfer of multiresistance genes mediated by plasmids across genera. Proc. Natl. Acad. Sci. USA.

[67] Ding C., Pan J., Jin M., Yang D., Shen Z., Wang J., Zhang B., Liu W., Fu J., Guo X. (2016). Enhanced uptake of antibiotic resistance genes in the presence of nanoalumina. Nanotoxicology.

[68] Guo M.T., Zhang G.S. (2017). Graphene oxide in the water environment could affect tetracycline-antibiotic resistance. Chemosphere.

[69] Wang X., Yang F., Zhao J., Xu Y., Mao D., Zhu X., Luo Y., Alvarez P. (2017). Bacterial exposure to ZnO nanoparticles facilitates horizontal transfer of antibiotic resistance genes. Nanoimpact.

[70] Zou W., Li X., Lai Z., Zhang X., Hu X., Zhou Q. (2016). Graphene oxide inhibits antibiotic uptake and antibiotic resistance gene propagation. ACS Appl. Mater. Interfaces.

[71] Huang H., Chen Y., Yang S., Zheng X. (2019). CuO and ZnO nanoparticles drive the propagation of antibiotic resistance genes during sludge anaerobic digestion: Possible role of stimulated signal transduction. Environ. Sci. Nano.

[72] Yu Y., Singh H., Tsitrin T., Bekele S., Lin Y.-H., Sikorski P., Moncera K.J., Torralba M.G., Morrow L., Wolcott R. (2021). Urethral catheter biofilms reveal plasticity in bacterial composition and metabolism and withstand host immune defenses in hypoxic environment. Front. Med..

[73] Michaelis C., Grohmann E. (2023). Horizontal gene transfer of antibiotic resistance genes in biofilms. Antibiotics.

[74] Afrasiabi S., Partoazar A. (2024). Targeting bacterial biofilm-related genes with nanoparticle-based strategies. Front. Microbiol..

[75] Shen M., Zhang Y., Zhu Y., Song B., Zeng G., Hu D., Wen X., Ren X. (2019). Recent advances in toxicological research of nanoplastics in the environment: A review. Environ. Pollut..

[76] Imran M., Das K.R., Naik M.M. (2019). Co-selection of multi-antibiotic resistance in bacterial pathogens in metal and microplastic contaminated environments: An emerging health threat. Chemosphere.

[77] Li J., Zhang K., Zhang H. (2018). Adsorption of antibiotics on microplastics. Environ. Pollut..

[78] Ashton K., Holmes L., Turner A. (2010). Association of metals with plastic production pellets in the marine environment. Mar. Pollut. Bull..

[79] Hartmann N.B., Rist S., Bodin J., Jensen L.H., Schmidt S.N., Mayer P., Meibom l., Baun A. (2017). Microplastics as vectors for environmental contaminants: Exploring sorption, desorption, and transfer to biota. Integr. Environ. Assess. Manag..

[80] Kirstein I.V., Kirmizi S., Wichels A., Garinfernandez A., Erler R., Loder M., Gerdts G. (2016). Dangerous hitchhikers? Evidence for potentially pathogenic *Vibrio* spp. on microplastic particles. Mar. Environ. Res..

[81] Mccormick A., Hoellein T.J., Mason S.A., Schluep J., Kelly J.J. (2014). Microplastic is an abundant and distinct microbial habitat in an urban river. Environ. Sci. Technol..

[82] Oberbeckmann S., Kreikemeyer B., Labrenz M. (2018). Environmental factors support the formation of specific bacterial assemblages on microplastics. Front. Microbiol..

[83] Parrish K., Fahrenfeld N. (2019). Microplastic biofilm in fresh-and wastewater as a function of microparticle type and size class. Environ. Sci. Water Res. Technol..

[84] Sorensen S.J., Bailey M.J., Hansen L.H., Kroer N., Wuertz S. (2005). Studying plasmid horizontal transfer in situ: A critical review. Nat. Rev. Microbiol..

[85] Ma Y., Wilson C.A., Novak J.T., Riffat R., Aynur S., Murthy S., Pruden A. (2011). Effect of various sludge digestion conditions on sulfonamide, macrolide, and tetracycline resistance genes and class I integrons. Environ. Sci. Technol..

[86] Munir M., Wong K., Xagoraraki I. (2011). Release of antibiotic resistant bacteria and genes in the effluent and biosolids of five wastewater utilities in Michigan. Water Res..

[87] Liu L., Gibson V., Huang X., Liu C., Zhu G. (2016). Effects of antibiotics on characteristics and microbial resistance of aerobic granules in sequencing batch reactors. Desalination Water Treat..

[88] Zhang M.Q., Yuan L., Li Z.H., Zhang H.C., Sheng G.P. (2019). Tetracycline exposure shifted microbial communities and enriched antibiotic resistance genes in the aerobic granular sludge. Environ. Int..

[89] Hoffman L.R., D’Argenio D.A., MacCoss M.J., Zhang Z., Jones R.A., Miller S.I. (2005). Aminoglycoside antibiotics induce bacterial biofilm formation. Nature.

[90] Song C., Sun X.F., Wang Y.K., Xia P.F., Yuan F.H., Li J.J., Wang S.G. (2016). Fate of tetracycline at high concentrations in enriched mixed culture system: Biodegradation and behavior. J. Chem. Technol. Biotechnol..

[91] Ibrahim A., Hiripitiyage Y., Peltier E., Sturm B.S. (2023). Biodegradation of aromatic compounds under hypersaline conditions: Comparing aerobic biofilm reactors with conventional activated sludge. Environ. Eng. Sci..

[92] Liu X., Sun S., Ma B., Zhang C., Wan C., Lee D.J. (2016). Understanding of aerobic granulation enhanced by starvation in the perspective of quorum sensing. Appl. Microbiol. Biotechnol..

[93] Xu H., Liu Y. (2011). Reduced microbial attachment by d-amino acid-inhibited AI-2 and EPS production. Water Res..

[94] Han H., Zhang K., Li G., Yu Y., Shi S., Liang C., Niu H., Zhuang W., Liu D., Yang P. (2023). Autoinducer-2: Its role in biofilm formation and L-Threonine production in *Escherichia coli*. Fermentation.

[95] Wang R., An Z., Fan L., Zhou Y., Su X., Zhu J., Zhang Q., Chen C., Lin H., Sun F. (2023). Effect of quorum quenching on biofouling control and microbial community in membrane bioreactors by *Brucella* sp. ZJ1. J. Environ. Manag..

[96] Bjarnsholt T., Jensen P.Ø., Burmølle M., Hentze M., Haagensen J.A.J., Hougen H.P., Calum H., Madsen K.G., Moser C., Molin S. (2005). *Pseudomonas aeruginosa* tolerance to tobramycin, hydrogen peroxide and polymorphonuclear leukocytes is quorum-sensing dependent. Microbiology.

[97] Shen Y., Gao S., Fan Q., Zuo J., Wang Y., Yi L., Wang Y. (2023). New antibacterial targets: Regulation of quorum sensing and secretory systems in zoonotic bacteria. Microbiol. Res..

[98] Zhu L., Chen T., Xu L., Zhou Z., Feng W., Liu Y., Chen H. (2020). Effect and mechanism of quorum sensing on horizontal transfer of multidrug plasmid RP4 in BAC biofilm. Sci. Total Environ..

[99] Yushina Y., Nasyrov N.A., Zaiko E., Grudistova M.A., Reshchikov M.D. (2023). Evaluating the effect of various types of disinfectants on bacterial biofilms. Theory Pract. Meat Process..

[100] Sun S., Tay Q.X.M., Kjelleberg S., Rice S.A., McDougald D. (2015). Quorum sensing-regulated chitin metabolism provides grazing resistance to Vibrio cholerae biofilms. ISME J..

[101] Flemming H.C. (2020). Biofouling and me: My Stockholm syndrome with biofilms. Water Res..

[102] Zhang Y., Love N., Edwards M. (2009). Nitrification in drinking water systems. Crit. Rev. Environ. Sci. Technol..

[103] Douterelo I., Dutilh B.E., Calero C., Rosales E., Martin K., Husband S. (2020). Impact of phosphate dosing on the microbial ecology of drinking water distribution systems: Fieldwork studies in chlorinated networks. Water Res..

[104] Fang W., Hu J.Y., Ong S.L. (2009). Influence of phosphorus on biofilm formation in model drinking water distribution systems. J. Appl. Microbiol..

[105] Chavant P., Martinie B., Meylheuc T., Bellon-Fontaine M.N., Hebraud M. (2002). *Listeria monocytogenes* LO28: Surface physicochemical properties and ability to form biofilms at different temperatures and growth phases. Appl. Environ. Microbiol..

[106] Labidi S., Jánosity A., Yakdhane A., Yakdhane E., Surányi B., Mohácsi-Farkas C., Kiskó G. (2023). Effects of pH, sodium chloride, and temperature on the growth of *Listeria monocytogenes* biofilms. Acta Aliment..

[107] Liu G., Zhang Y., Liu X., Hammes F., Liu W.T., Medema G., Wessels P., Van der Meer W.G. (2020). 360-Degree distribution of biofilm quantity and community in an operational unchlorinated drinking water distribution pipe. Environ. Sci. Technol..

[108] Kilb B., Lange B., Schaule G., Flemming H.C., Wingender J. (2003). Contamination of drinking water by coliforms from biofilms grown on rubber-coated valves. Int. J. Hyg. Environ. Health.

[109] Shan L., Zheng W., Xu S., Xu S., Zhu Z., Pei Y., Bao X., Yuan Y. (2024). Effect of household pipe materials on formation and chlorine resistance of the early-stage biofilm: Various interspecific interactions exhibited by the same microbial biofilm in different pipe materials. Arch. Microbiol..

[110] Uppuluri P. (2022). A simple method for growth of Candida albicans biofilms under continuous media flow and for recovery of biofilm dispersed cells. Methods Mol. Biol..

[111] Simoes L.C., Simoes M., Vieira M.J. (2010). Influence of the diversity of bacterial isolates from drinking water on resistance of biofilms to disinfection. Appl. Environ. Microbiol..

[112] Moreira J.M., Teodosio J.S., Silva F.C., Simões M., Melo L.F., Mergulhão F.J. (2013). Influence of flow rate variation on the development of *Escherichia coli* biofilms. Bioprocess Biosyst. Eng..

[113] Fish K., Osborn A.M., Boxall J.B. (2017). Biofilm structures (EPS and bacterial communities) in drinking water distribution systems are conditioned by hydraulics and influence discolouration. Sci. Total Environ..

[114] Yan J., Nadell C.D., Bassler B.L. (2017). Environmental fluctuation governs selection for plasticity in biofilm production. ISME J..

[115] Engemann C.A., Keen P.L., Knapp C.W., Hall K.J., Graham D.W. (2008). Fate of tetracycline resistance genes in aquatic systems: Migration from the water column to peripheral biofilms. Environ. Sci. Technol..

[116] Siedlecka A., Wolf-Baca M., Piekarska K. (2021). Microbial communities of biofilms developed in a chlorinated drinking water distribution system: A field study of antibiotic resistance and biodiversity. Sci. Total Environ..

[117] Zhang J., Li W., Chen J., Wang F., Qi W., Li Y. (2019). Impact of disinfectant on bacterial antibiotic resistance transfer between biofilm and tap water in a simulated distribution network. Environ. Pollut..

[118] Kimbell L.K., Lamartina E.L., Kappell A.D., Huo J., Wang Y., Newton R.J., McNamara P.J. (2021). Cast iron drinking water pipe biofilms support diverse microbial communities containing antibiotic resistance genes, metal resistance genes, and class 1 integrons. Environ. Sci. Water Res. Technol..

[119] Rilstone V., Vignale L., Craddock J., Cushing A., Filion Y., Champagne P. (2021). The role of antibiotics and heavy metals on the development, promotion, and dissemination of antimicrobial resistance in drinking water biofilms. Chemosphere.

[120] Lakshmi A.N., Bhuyan A.D., Pasupulety L. (2024). Effect of corrosion media on biofilm detachment and the corrosion mechanism of Serratia marcescens on carbon steel in river water. J. Bio-Tribo-Corros..

[121] Batinovic S., Wassef F., Knowler S.A., Rice D.T., Stanton C.R., Rose J., Tucci J., Nittami T., Vinh A., Drummond G.R. (2019). Bacteriophages in natural and artificial environments. Pathogens.

[122] Śliwka P., Ochocka M., Skaradzinska A. (2022). Applications of bacteriophages against intracellular bacteria. Crit. Rev. Microbiol..

[123] Figueiredo C.M., Malvezzi Karwowski M.S., Da Silva Ramos R.C.P., de Oliveira N.S., Peña L.C., Carneiro E., Freitas de Macedo R.E., Rosa E.A.R. (2021). Bacteriophages as tools for biofilm biocontrol in different fields. Biofouling.

[124] Wan Q., Bao H., Zhang H., Zhu S., Ran W., Yan Z. (2022). Research progress in the interactions between bacteriophages and bacterial biofilms. Chin. J. Anim. Infect. Dis..

[125] Topka-Bielecka G., Dydecka A., Necel A., Bloch S., Nejman-Faleńczyk B., Węgrzyn G., Węgrzyn A. (2021). Bacteriophage-derived depolymerases against bacterial biofilm. Antibiotics.

[126] Arciola C.R., Campoccia D., Montanaro L. (2018). Implant infections: Adhesion, biofilm formation and immune evasion. Nat. Rev. Microbiol..

[127] Yu Z., Schwarz C., Zhu L., Chen L., Shen Y., Yu P. (2021). Hitchhiking behavior in bacteriophages facilitates phage infection and enhances carrier bacteria colonization. Environ. Sci. Technol..

[128] Zhang B., Yu P., Wang Z., Alvarez P.J.J. (2020). Hormetic promotion of biofilm growth by polyvalent bacteriophages at low concentrations. Environ. Sci. Technol..

[129] Secor P.R., Burgener E.B., Kinnersley M., Jennings L.K., Roman-Cruz V., Popescu M., Van Belleghem J.D., Haddock N., Copeland C., Michaels L.A. (2020). Pf bacteriophage and their impact on *pseudomonas* virulence, mammalian immunity, and chronic infections. Front. Immunol..

[130] Binh C.T., Heuer H., Kaupenjohann M., Jennings L.K., Roman-Cruz V., Popescu M., Van Belleghem J.D., Haddock N., Copeland C., Michaels L.A. (2008). Piggery manure used for soil fertilization is a reservoir for transferable antibiotic resistance plasmids. FEMS Microbiol. Ecol..

[131] Brussow H., Hendrix R.W. (2002). Phage genomics: Small is beautiful. Cell.

[132] Li P.Z., Chen B.B., Song Z.J., Song Y., Yang Y., Ma P., Wang H., Ying J., Ren P., Yang L. (2012). Bioinformatic analysis of the *Acinetobacter baumannii* phage AB1 genome. Gene.

[133] Gabashvili E., Osepashvili M., Koulouris S., Ujmajuridze L., Tskhitishvili Z., Kotetishvili M. (2020). Phage transduction is involved in the intergeneric spread of antibiotic resistance-associated *bla (CTX-M)*, *mel*, and *tetM* loci in natural populations of some human and animal bacterial pathogens. Curr. Microbiol..

[134] Dion M.B., Oechslin F., Moineau S. (2020). Phage diversity, genomics and phylogeny. Nat. Rev. Microbiol..

[135] Shousha A., Awaiwanont N., Sofka D., Smulders F.J., Paulsen P., Szostak M.P., Humphrey T., Hilbert F. (2015). Bacteriophages isolated from chicken meat and the horizontal transfer of antimicrobial resistance genes. Appl. Environ. Microbiol..

[136] Haaber J., Leisner J.J., Cohn M.T., Catalan-Moreno A., Nielsen J.B., Westh H., Penadés J.R., Ingmer H. (2016). Bacterial viruses enable their host to acquire antibiotic resistance genes from neighbouring cells. Nat. Commun..

[137] Aggarwala V., Liang G., Bushman F.D. (2017). Viral communities of the human gut: Metagenomic analysis of composition and dynamics. Mob. DNA.

[138] Shkoporov A.N., Hill C. (2019). Bacteriophages of the human gut: The “known unknown” of the microbiome. Cell Host Microbe.

[139] Moon K., Jeon J.H., Kang I., Park K.S., Lee K., Cha C.J., Lee S.H., Cho J.C. (2020). Freshwater viral metagenome reveals novel and functional phage-borne antibiotic resistance genes. Microbiome.

[140] Lang A.S., Zhaxybayeva O., Beatty J.T. (2012). Gene transfer agents: Phage-like elements of genetic exchange. Nat. Rev. Microbiol..

[141] Karaolis D.K., Johnson J.A., Bailey C.C., Boedeker E.C., Kaper J.B., Reeves P.R. (1998). A vibrio cholerae pathogenicity island associated with epidemic and pandemic strains. Proc. Natl. Acad. Sci. USA.

[142] Faruque S.M., Mekalanos J.J. (2003). Pathogenicity islands and phages in Vibrio cholerae evolution. Trends Microbiol..

[143] Santoriello F.J., Michel L., Unterewger D., Pukatzki S. (2020). Pandemicvibrio cholerae shuts down site-specific recombination to retain an inter bacterial defence mechanism. Nat. Commun..

[144] Debroas D., Siguret C. (2019). Viruses as key reservoirs of antibiotic resistance genes in the environment. ISME J..

[145] Fillol-Salom A., Alsaadi A., Sousa J.A.M., Zhong L., Foster K.R., Rocha E.P.C., Penadés J.R., Ingmer H., Haaber J. (2019). Bacteriophages benefit from generalized transduction. PLoS Pathog..

[146] Jain P., Hsu T., Arai M., Biermann K., Thaler D.S., Nguyen A., González P.A., Tufariello J.M., Kriakov J., Chen B. (2014). Specialized transduction designed for precise high-throughput unmarked deletions in *Mycobacterium tuberculosis*. mBio.

[147] Tufariello J.M., Malek A.A., Vilchezen C., Cole L.E., Ratner H.K., González P.A., Jain P., Hatfull G.F., Larsen M.H., Jacobs W.R. (2014). Enhanced specialized transduction using recombineering in *Mycobacterium tuberculosis*. mBio.

[148] Argov T., Azulay G., Pasechnek A., Stadnyuk O., Ran-Sapir S., Borovok I., Sigal N., Herskovits A.A. (2017). Temperate bacteriophages as regulators of host behavior. Curr. Opin. Microbiol..

[149] Paez-Espino D., Eloe-Fadrosh E.A., Pavlopoulos G.A., Thomas A.D., Huntemann M., Mikhailova N., Rubin E., Ivanova N.N., Kyrpides N.C. (2016). Uncovering earth’s virome. Nature.

[150] Touchon M., de Sousa J.A.M., Rocha E.P. (2017). Embracing the enemy: The diversification of microbial gene repertoires by phage-mediated horizontal gene transfer. Curr. Opin. Microbiol..

[151] Ross J., Topp E. (2015). Abundance of antibiotic resistance genes in bacteriophage following soil fertilization with dairy manure or municipal biosolids, and evidence for potential transduction. Appl. Environ. Microbiol..

[152] Fard R.M.N., Barton M.D., Heuzenroeder M.W. (2011). Bacteriophage-mediated transduction of antibiotic resistance in *enterococci*. Lett. Appl. Microbiol..

[153] Madsen J.S., Burmølle M., Hansen L.H., Sørensen S.J. (2012). The interconnection between biofilm formation and horizontal gene transfer. FEMS Immunol. Med. Microbiol..

[154] Bowler P., Murphy C., Wolcott R. (2020). Biofilm exacerbates antibiotic resistance: Is this a current oversight in antimicrobial stewardship?. Antimicrob. Resist. Infect. Control.

[155] Rao R.S., Karthika R.U., Singh S., Shashikala P., Kanungo R., Jayachandran S., Prashanth K. (2008). Correlation between biofilm production and multiple drug resistance in imipenem resistant clinical isolates of *Acinetobacter baumannii*. Indian J. Med. Microbiol..

[156] Khoshnood S., Sadeghifard N., Mahdian N., Heidary M., Mahdian S., Mohammadi M., Maleki A., Haddadi M.H. (2023). Antimicrobial resistance and biofilm formation capacity among *Acinetobacter baumannii* strains isolated from patients with burns and ventilator-associated pneumonia. J. Clin. Lab. Anal..

[157] Bardbari A.M., Arabestani M.R., Karami M., Keramat F., Alikhani M.Y., Bagheri K.P. (2017). Correlation between the ability of biofilm formation with their responsible genes and MDR patterns in clinical and environmental *Acinetobacter baumannii* isolates. Microb. Pathog..

[158] Qi L., Li H., Zhang C., Liang B., Li J., Wang L., Du X., Liu X., Qiu S., Song H. (2016). Relationship between antibiotic resistance, biofilm formation, and biofilm-specific resistance in *Acinetobacter baumannii*. Front. Microbiol..

[159] Rodrigues Perez L.R. (2015). *Acinetobacter baumannii* displays inverse relationship between meropenem resistance and biofilm production. J. Chemother..

[160] Wang X., Tang Y., Yue X., Wang S., Yang K., Xu Y., Shen Q., Friman V.P., Wei Z. (2024). The role of rhizosphere phages in soil health. FEMS Microbiol. Ecol..

[161] Sobecky P.A., Hazen T H. (2009). Horizontal gene transfer and mobile genetic elements in marine systems. Methods Mol. Biol..

[162] Eggers C.H., Gray C.M., Preisig A.M., Glenn D.M., Pereira J., Ayers R.W., Alshahrani M., Acabbo C., Becker M.R., Bruenn K.N. (2016). Phage-mediated horizontal gene transfer of both prophage and heterologous DNA by ϕBB-1, a bacteriophage of *Borrelia burgdorferi*. Pathog. Dis..

[163] Mancini K., Cao I., Ge C., Mu E., Zhu R., Mathur V. (2024). Investigating the role of phage mediated HGT in increasing bacterial virulence. Bios.

[164] Uyar N.Y., Aya M., Kocagz A.S. (2024). Antibiotic resistance profile of *Pseudomonas aeruginosa* strains isolated from blood culture of patients in intensive care units. J. Crit. Care.

