# Supplementary material for: Review of the Presence and Phage-Mediated Transfer of ARGs in Biofilms
Source: Microorganisms. 2025 Apr 26;13(5):997. doi: 10.3390/microorganisms13050997 (PMC12114417; doi:10.3390/microorganisms13050997)
Supplement: Supplementary file 1 [file microorganisms-13-00997-s001.zip › microorganisms-3550605-supplementary.pdf]

Table S1. Classification of the ARGs

ARGs	Classification	Resistance Mechanism	Resistance Category	Characteristic Description
tetA, tetB, tetL, tetG, tetZ	Tetracycline	Efflux pump	Tetracycline antibiotics	Encodes an efflux pump protein that expels tetracycline antibiotics from the cell
tetO	Tetracycline	Ribosomal protection protein	Tetracycline antibiotics	Encodes a ribosomal protection protein that prevents tetracycline from binding to the ribosome
tetW, tetM, tetQ	Tetracycline	Ribosomal protection protein	Tetracycline antibiotics	Encodes a ribosomal protection protein that protects the ribosome from tetracycline inhibition
sulI, sulII, sulIII	Sulfonamide	Dihydropteroate synthase variant	Sulfonamide antibiotics	Encodes a variant dihydropteroate synthase with reduced binding affinity for sulfonamides
qnrS	Quinolone	DNA gyrase protection protein	Quinolone antibiotics	Encodes a DNA gyrase protection protein that prevents quinolones from binding to DNA gyrase
strA, strB	Aminoglycoside	Aminoglycoside-modifying enzyme	Aminoglycoside antibiotics	Encodes an aminoglycoside-modifying enzyme that inactivates the antibiotic
blaAMP	β-Lactam	β-Lactamase	β-Lactam antibiotics	Encodes a β-lactamase that hydrolyzes β-lactam antibiotics
blaTEM	β-Lactam	β-Lactamase	β-Lactam antibiotics	Encodes a TEM-type β-lactamase that hydrolyzes β-lactam antibiotics
blaOXA	β-Lactam	β-Lactamase	β-Lactam antibiotics	Encodes an OXA-type β-lactamase that hydrolyzes β-lactam antibiotics
blaCTX-M	β-Lactam	β-Lactamase	β-Lactam antibiotics	Encodes a CTX-M-type β-lactamase that hydrolyzes β-lactam antibiotics
AmpC	β-Lactam	β-Lactamase	β-Lactam antibiotics	Encodes an AmpC-type β-lactamase that hydrolyzes β-lactam antibiotics
ermX, ermB, ermF	Macrolide	23S rRNA methyltransferase	Macrolide antibiotics	Encodes a 23S rRNA methyltransferase that modifies the ribosome, preventing macrolide binding

The abbreviations of the terms:

1. ARGs - Antibiotic resistance genes

2. MPs – Microplastics
3. MGEs - mobile genetic elements
4. HGT - horizontal gene transfer
5. QS - quorum sensing
6. VGT - Vertical gene transfer
7. ARB - antibiotic-resistant bacteria
8. AMR - antimicrobial resistance
9. MCR-1 - mobilized colistin resistance gene 1
10. ROS - reactive oxygen species
11. GO - Graphene oxide
12. PE – polyethylene
13. PP – polypropylene
14. PS – polystyrene
15. PVC - polyvinyl chloride
16. MRGs - metal resistance genes
17. AGS - aerobic granular sludge
18. EPS - extracellular polymeric substances
19. AIs – Autoinducers
20. AHLs - acyl homoserine lactones
21. BOM - biodegradable organic matter
22. UV – ultraviolet
23. VAPGHs - virion-associated peptidoglycan hydrolases
24. TSP - tail spike protein
25. LPS – lipopolysaccharides
26. eDNA - extracellular DNA
27. VPI - Vibrio pathogenicity island
28. sub-MICs - sub-minimum inhibitory concentrations
29. XDR - extensive drug-resistant
30. MDR - multi-drug-resistant
